# Excretion kinetics of 1,3-dichlorobenzene and its urinary metabolites after controlled airborne exposure in human volunteers

**DOI:** 10.1007/s00204-023-03447-x

**Published:** 2023-01-30

**Authors:** T. Schettgen, J. Bertram, J. Krabbe, R. Christoforou, M. Schweiker, A. Esser, M. Möller, P. Ziegler, T. Kraus

**Affiliations:** Institute for Occupational, Social and Environmental Medicine, Medical Faculty, RWTH Aachen University, Pauwelsstrasse 30, 52074 Aachen, Germany

**Keywords:** Dichlorocatechol, Dichlorphenol, Human biomonitoring, Silicone, Toxicokinetics

## Abstract

The solvent 1,3-dichlorobenzene (1,3-DCB) is formed during thermal decomposition of the initiator 2,4-dichlorobenzoylperoxide in the production of silicone rubber with potential exposure of production workers as shown in previous works. Despite a threshold limit value (MAK value) of 2 ppm in air, there are currently no data about the corresponding internal exposure that would allow for the derivation of a biological limit value. In the present study, we have investigated the absorption of 1,3-DCB and urinary kinetics of its metabolites in 10 human volunteers after controlled inhalative exposure. Due to the strong odour of 1,3-DCB, a subjective evaluation of odour nuisance was also performed. Ten male human volunteers (23–36 yrs.) were exposed 6 h/day to a concentration of 0.7 ppm and 1.5 ppm in the Aachen workplace simulation laboratory (AWSL) with one week between each experiment. In order to investigate potential dermal absorption, the volunteers were exposed to 1.5 ppm wearing a suitable filter mask that prevented inhalative exposure in a third exposure. 1,3-DCB in blood was measured after 3 and 6 h exposure and the urinary metabolites 3,5-dichlorocatechol (3,5-DCC), 2,4-dichlorophenol (2,4-DCP) and 3,5-dichlorophenol (3,5-DCP) were measured over 24 h after exposure via LC/MS/MS. There were clear dose–response relations for all investigated parameters. The maximum excretion of the metabolites was reached at the end of exposure and corresponded to 5.2 ± 0.7 mg/g crea, 1.5 ± 0.35 mg/g crea and 0.07 ± 0.011 mg/g crea at 0.7 ppm and to 12.0 ± 3 mg/g crea, 3.5 ± 1.1 mg/g crea and 0.17 ± 0.05 mg/g crea at 1.5 ppm for 3,5-DCC, 2,4-DCP and 3,5-DCP, respectively. The use of filter masks decreased the internal exposure for about 85–90%, indicating substantial dermal absorption. Odour perception did not show a dose–response, probably due to fast olfactory adaption. The human study presented here provides an excellent basis for deriving a biological limit value for 1,3-DCB.

**Supplementary Information:**

The online version contains supplementary material available at 10.1007/s00204-023-03447-x.

## Introduction

1,3-dichlorobenzene (1,3-DCB) is a clear liquid with a boiling point of 173 °C and a strong odour. It has been shown to increase serum cholesterol levels as well as liver and kidney weights at doses above 37 mg/kg bw in a 90-day feeding trial on Sprague–Dawley rats (McCauley et al. 1995). Furthermore, a phenobarbital-like induction of glucuronyltransferase I as well as metabolising liver enzymes was observed in rats in a 28-day study at the lowest dose given (4 mg/kg bw). This enzyme induction also influences the endocrine system, affecting the thyroid at doses above 35 mg/kg bw in rats. Tests for a genotoxic and carcinogenic potential of 1,3-DCB showed inconclusive results; thus, it is not classified as a carcinogen by the Senate Commission for the investigation of health hazards at the workplace of the Deutsche Forschungsgemeinschaft (DFG), in contrast to the isomeric 1,4-dichlorobenzene (carcinogen group 4) (summarised in DFG 2013).

The DFG has evaluated a threshold limit value of 2 ppm (= 12 mg/m^3^) in workplace air for 1,3-DCB to prevent workers from potential health hazards (DFG 2022). In our previous investigation, we discovered that workers from the silicone rubber industry are exposed to quite high levels of 1,3-DCB by human biomonitoring of the corresponding metabolites 3,5-dichlorocatechol as well as 2,4- and 3,5-dichlorophenol in post-shift urine with maximum levels of 26.9 mg/L, 7.7 mg/L and 390 µg/L, respectively (Schettgen et al. 2022). 1,3-DCB is formed during thermal decomposition of the initiator 2,4-dichlorobenzoylperoxide (2,4-DCBP) that is used in the production of silicone rubber (Herkert et al. 2018). The interpretation of these metabolite levels determined in urine of the workers was difficult, as a biological limit value or quantitative data on the absorption, distribution, metabolism and excretion of 1,3-DCB in humans are not yet available.

In contrast, the DFG has evaluated biological limit values (BAT values) for the well-investigated isomers 1,2-dichlorobenzene (140 µg 1,2-dichlorobenzene/L blood right after exposure or 150 mg/g creatinine for the sum of the urinary metabolites 3,4 and 4,5-dichlorocatechol post-shift or after several shifts, corresponding to an exposure of 10 ppm) and 1,4-dichlorobenzene (10 mg/L for the urinary metabolite 2,5-dichlorophenol post-shift or after several shifts, corresponding to an exposure of 2 ppm) (DFG 2022).

**Fig. 1:**
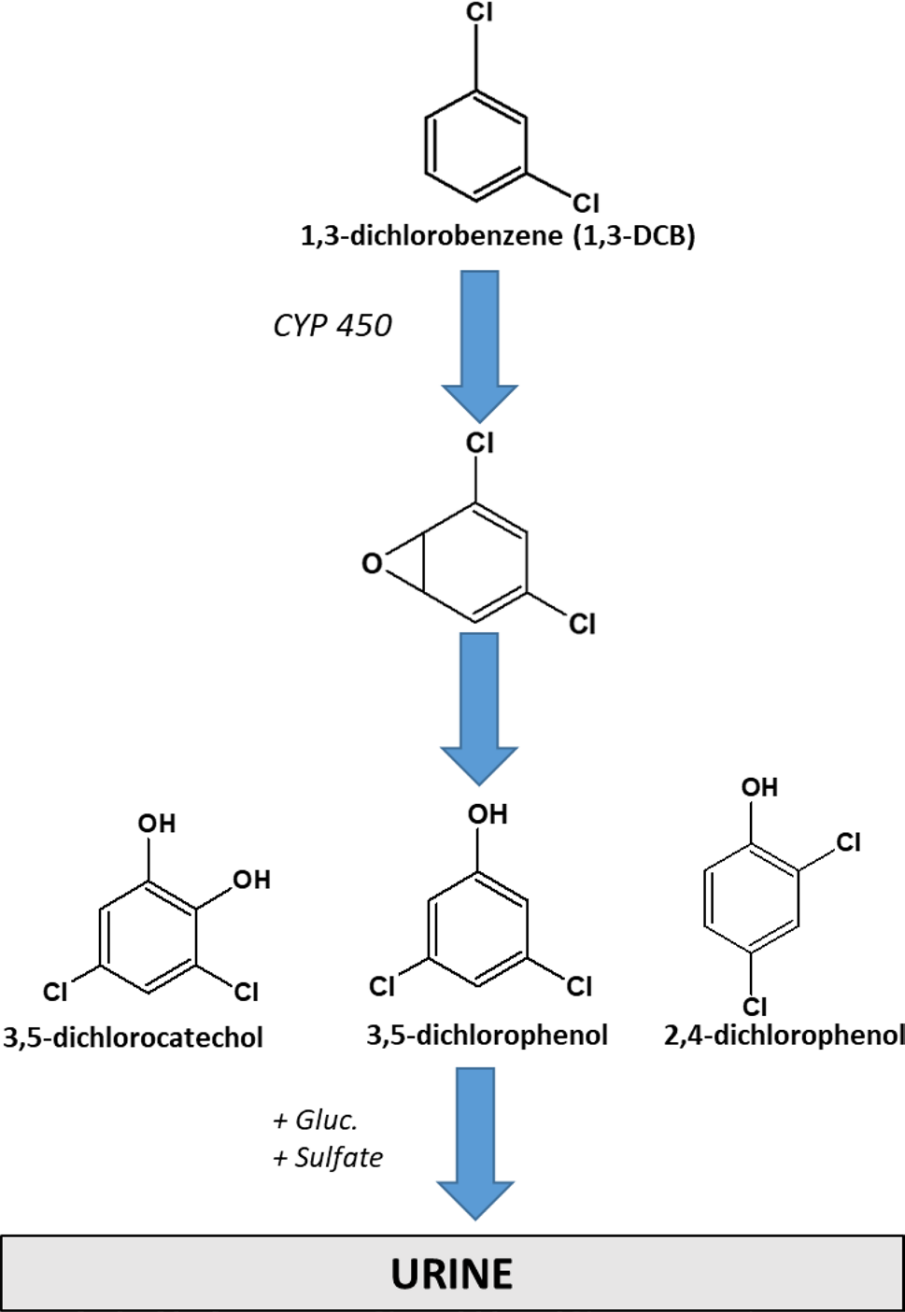
Formation of phenolic metabolites from 1,3-DCB in humans

The current study was aimed to fill this gap and to determine the excretion kinetics of 1,3-DCB and its urinary metabolites in volunteers after controlled inhalative exposure in order to set the basis for the evaluation of a biological limit value for 1,3-DCB. As 1,3-DCB has a very strong and disturbing odour, data on the subjective evaluation of odour nuisance of the volunteers were also acquired using self-reported questionnaires. With the help of our combined data, the exposure situation of the workers in the silicone industry might be evaluated. A scheme of the assumed metabolism of 1,3-DCB with the formation of the phenols 2,4- and 3,5-dichlorophenol as well as 3,5-dichlorocatechol is depicted in Fig. 1.

To our knowledge, data on controlled human exposure to 1,3-DCB and metabolite excretion as well as subjective evaluation of odour nuisance have not been reported before.

## Materials and methods

### Experimental design

In October 2021, ten male healthy, never-smoking volunteers (age: 23–36 years, body weight: 59–143 kg) were exposed to defined air concentrations of 1,3-DCB in the Aachen workplace simulation laboratory. All participants were never smokers and had no history of asthma or any other lung or cardiac disease. An overview on the biometric and anamnestic data of the volunteers is given in Table 1.

**Table 1:** Anamnestic data of the male human volunteers

Volunteer	Age [years]	Weight [kg]	Height [cm]	BMI	Beard
1	23	80	180	24.7	
2	24	81	179	25.3	
3	36	143	190	39.6	+ +
4	22	81	175	26.4	
5	27	88	186	25.4	
6	29	67	174	22.1	+
7	31	106	186	30.6	+
8	24	76	171	26.0	
9	23	59	172	19.9	
10	23	67	184	19.8	

The target concentration for the exposures to 1,3-DCB was 0.70 ppm and 1.5 ppm, for which the volunteers were exposed on one day for 6 h (2 × 3 h with a short interruption of 0.5 h for lunch outside the laboratory). These levels are below the current air limit value of 2 ppm (evaluated for a work shift of 8 h by the DFG), which is considered safe. In order to ensure comparable conditions for each person, the volunteers were dressed uniformly in surgical scrubs (short sleeves and long legs) during exposure, also avoiding possible absorption on their street clothes that could lead to a reservoir of 1,3-DCB and odour nuisance.

To investigate the effectiveness of personal protective measures as well as a potential dermal absorption of 1,3-DCB (1,2- and 1,4-dichlorobenzene are marked as skin resorptive “H”), a third exposure of the volunteers was carried out at 1.5 ppm with all volunteers wearing organic vapour/dust respirator masks (3 M, 4251 +) that should prevent inhalation of 1,3-DCB according to the manufacturers’ specification.

Each exposure was carried out with two groups of n = 5 volunteers in the workplace simulation laboratory with one week between each exposure in order to eliminate the 1,3-DCB metabolites. During the experiments, all volunteers were left at rest in the laboratory without extra physical activity. This study started with the high exposure (1.5 ppm), followed by the low exposure (0.70 ppm) in the following week and finally the exposure at 1.5 ppm with the wearing of the protective masks. All experiments were carried out under room temperature (app. 20–22 °C) and ambient humidity (data not recorded).

The volunteers provided a blood sample after 3 h (before lunchbreak) and at the end of the whole 6-h exposure that was immediately transferred to a gas-tight glass vial for the following headspace determination of 1,3-DCB in blood. Furthermore, each volunteer provided one urine sample before the exposure (t_0_) and consecutively collected their urine samples over the following 24 h. The time of collection of the urine sample was recorded by the volunteer. Urine volume for each void was determined by weighing difference of the filled and empty urine containers, assuming urine density of 1.00 g/mL. The urine samples provided were aliquoted in 10-ml-aliquots and stored frozen at–20 °C until analysis. Creatinine content of the urine samples was determined photometrically using the Jaffé method (Larsen 1972). This study was approved by the Ethics committee of the RWTH Aachen University (EK 403/20).

### Chemicals

1,3-dichlorobenzene (98%) was purchased from Aldrich (Steinheim, Germany). The metabolites 3,5-dichlorocatechol (97%), 3,5-dichlorophenol (Pestanal grade) and 2,4-dichlorophenol (Pestanal grade) were also purchased from Aldrich (Steinheim, Germany). The isotope labelled internal standards D_3_-3,5-dichlorophenol (98,5% D_3_) and D_3_-2,4-dichlorophenol (98,5% D_3_) were provided from CDN Isotopes (Pointe Claire, Quebec, Canada). D_3_-2,4-dichlorobenzoic acid (98,5% D_3_) was used as internal standard for the quantification of 3,5-dichlorocatechol and was also purchased from CDN Isotopes (Pointe Claire, Quebec, Canada).

Formic acid (100%) and water (LC/MS grade) was supplied by Merck (Darmstadt, Germany). Acetonitrile (HPLC grade) was purchased from J.T. Baker (Germany). Ammonium formate was supplied by Fluka (Buchs, Suisse).

### Generation of a stable 1,3-DCB concentration in the Aachen workplace simulation laboratory (AWSL)

**Fig. 2:**
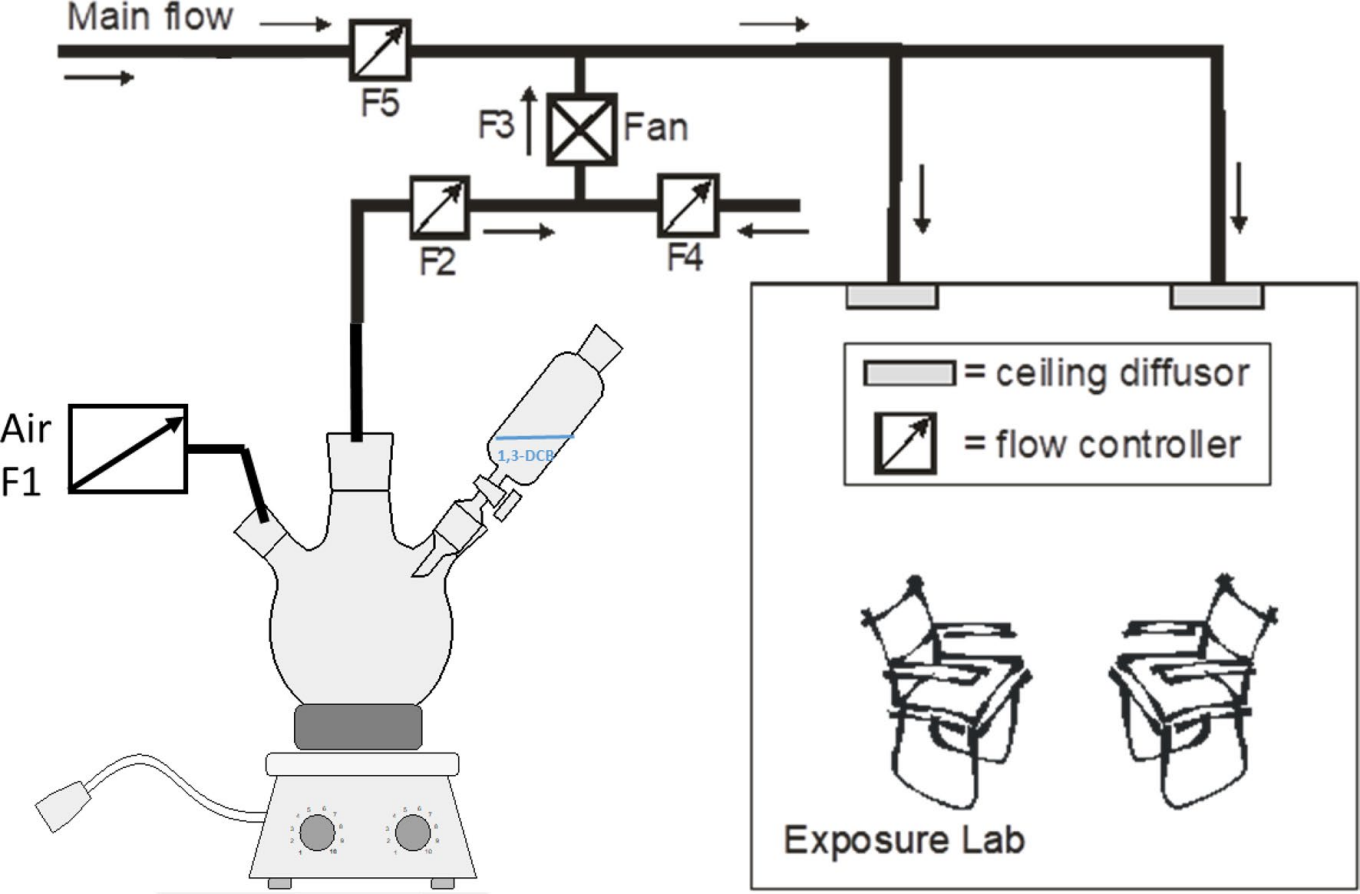
Scheme of the Aachen Workplace Simulation Laboratory (AWSL) with the generation of 1,3-dichlorbenzene vapour

The AWSL consists of two different units: the control room, in which the 1,3-DCB vapours were generated and adjusted and the exposure room, in which the test subjects are exposed (Fig. 2). Both units are connected by a ventilation system that regulates the 1,3-DCB concentration in the exposure room. The exposure room has a volume of about 41 m^3^ (3.5 m × 4.7 m × 2.5 m) and is suitable for up to 6 subjects. Under normal conditions, the room is ventilated with an air flow of 550 m^3^/h. The flow into the chamber can be manually adjusted to obtain the desired concentration. The generated 1,3-DCB vapour containing air enters the exposure room via four ceiling diffusors with vortex flow which ensures a homogeneous vapour distribution throughout the room. Figure 2 shows a scheme of the ventilation system. Using the (manually controlled) different flow controllers, we were able to dilute the generated 1,3-DCB vapour to a stable target concentration. The 1,3-DCB vapour was generated using a three-necked flask with a large volume (2 L) that was placed in a heating mantle heated at 200 °C. One of the necks was connected to a syringe pump providing a continuous flow of pure 1,3-DCB to the bottom of the neck (depicted as a dropping funnel in Fig. 1). The second neck was connected to a flow-regulated air supply (F1), while the third neck was connected via a heated pipe to the variable flow controller F2, which was used to adjust the concentration in the exposure room. A picture of the vapour generating flask is shown in the Supplement to this manuscript (Figure S1).

**Fig. 3:**
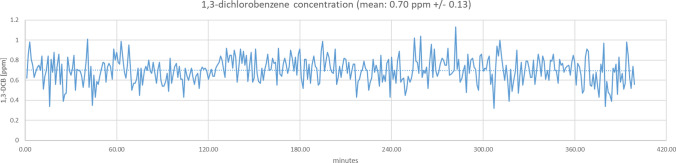
Time course of the 1,3-dichlorobenzene concentration in the exposure lab at the lower concentration as provided by the Gasmet FTIR

The concentration of 1,3-DCB in the exposure chamber was continuously monitored by a Gasmet D × 4040 FTIR portable gas analyser previously calibrated for 1,3-DCB (Gasmet Technologies GmbH, Karlsruhe, Germany). The limit of quantification of the Gasmet analyser was 0.5 ppm as stated by the manufacturers’ specifications. Furthermore, the stability of the exposure was additionally checked by gas monitoring via absorption on Tenax and subsequent analysis via GC/MS. The concentrations determined by the Gasmet analyser and the Tenax measurements showed good accordance (data not shown). For each target concentration, the initial adjustments of air flow parameters, heating mantle temperature as well as syringe pump and air supply were checked in advance without volunteers in the exposure chamber, so that only manual fine adjustments were necessary under experimental conditions. The exemplary time course of 1,3-DCB concentrations in the exposure chamber for a low concentration experiment (0.7 ppm) is depicted in Fig. 3.

### Determination of 1,3-DCB in blood by headspace GC/MS

1 ml of whole blood was transferred into 20-ml headspace vials with Teflon-laminated butyl rubber septa. 1,3-DCB was determined with a TurboMatrix HS 40 Trap headspace injector (Perkin-Elmer, Rodgau, Germany) working in multiple headspace extraction mode (Kolb and Ettre 1991). This headspace injector is directly coupled to a Thermo Trace GC Ultra/Thermo DSQ II GC/MS system (Thermo Fischer, Waltham, MA, USA) working in single ion monitoring mode (SIM mode) using m/z 146 as specific mass. The temperature of the headspace vial was 80 °C for 50 min with a fivefold multiple headspace extraction. The starting temperature of the GC was 150 °C for 1 min, increased by 10 °C/min until a final temperature of 230 °C was held for 0.01 min. Chromatography was performed on a Rxi-624 Sil MS column (60 m × 0,31 mm ID; 1,8 µm film thickness; Restek, Bad Homburg, Germany) using Helium 5.0 as a carrier gas with a constant pressure of 137.9 kPa.

Quantification was performed using an external calibration in sheep blood in the range of 0.5–20 µg/L. The limit of quantification–based on a signal-to-noise ratio of 6 was determined to be 0.1 µg/L.

### Determination of urinary metabolites of 1,3-DCB

500 µL of urine is pipetted into a 1.8-ml glass vial for HPLC with screw top and Teflon-lined cap. 500 µL of 100 mM ammonium acetate buffer (pH 5.0, adjusted with acetic acid) is added. 10 µL of the solution of the Internal Standards in water (D3-2,4-DCBA, 250 mg/L; D3-2,4-DCP, 50 mg/L; D3-3,5-DCP, 5 mg/L) and 5 µL of β-glucuronidase/arylsulphatase from Helix pomatia (Roche Diagnostics, Mannheim, Germany) are added, and the glass vial is shortly vortexed and incubated at 37 °C in an oven overnight. 30 µL of this solution is injected into the online SPE-LC/MS/MS system.

### Online SPE-LC/MS/MS

Chromatographic separation was carried out on a LC system (Agilent Technologies 1290 Infinity II series) consisting of a degasser, two binary pumps and an autosampler equipped with a six-port divert valve. Chromatography was performed using water, adjusted to pH 2.5 using formic acid as mobile phase A and acetonitrile as mobile phase B. Briefly, 30 µL of the sample is injected onto a LiChrospher^®^ RP-8 ADS column (25 µm; 24 × 4 mm from Merck (Darmstadt, Germany)) with a constant flow of 0.95 ml min-1, starting with conditions of 88% A and 12% B (acetonitrile) for online matrix depletion and analyte enrichment. After 5 min under these conditions, the six-port valve switches and the analyte is flushed back onto the analytical column (Phenomenex Kinetex F5, 150 × 4.6 mm, 2.6 µM, 100 Å) using starting conditions of 50% A and 50% B (acetonitrile) at a flow rate of 0.4 ml min-1.

During separation on the analytical column, the LiChrospher® RP-8 ADS column is washed and re-equilibrated again. The full gradient programme for both pumps and switching times for the six-port valve are summarised in the Supplement (Table S1). A precolumn filter (0.5 µm, Supelco) and a Security guard column (Kinetex F5, 4 × 3 mm, Phenomenex) was placed in front of the analytical column for protection against particles. The temperature of the analytical column was kept at 35 °C using a column thermostat.

The tandem mass spectrometric detection was performed on a Sciex API 4500 LC/MS/MS system (Sciex, Darmstadt, Germany) in ESI-negative mode. The ion source conditions were identical for all analytes with an electrospray needle voltage of–4.500 V in the negative ion mode and source temperature of 550 °C. Nebuliser gas (“gas 1”) and desolvation gas (“gas 2”) were set to 16 and 0 units. Curtain gas and collision gas were set to 40 psi and 10 arbitrary units, respectively. The system was operated in multiple reaction monitoring mode (MRM) with mass spectrometry parameters previously optimised using continuous flow injection of standards (5 ng ml^-1^) using the syringe pump of the instrument. Retention time (RT), collision energies (CE) and collision cell exit potentials (CXP) for the analytes and internal standards are summarised in the Supplement (Table S2). A six-port valve connected to the instrument was used to control the flow in the mass spectrometer, so that only the fraction of interest (11–14.5 min, cf. Table S2) from the LC system is transferred to the mass spectrometer to avoid unnecessary contamination. Eight calibration standards with concentrations ranging from 50 to 10.000 µg/L (3,5-DCC) 20 – 4.000 µg/L (2,4-DCP) and 2–800 µg/L (3,5-DCP) were prepared by diluting spiking solutions with pooled urine (creatinine content: 0.56 g/L). These calibration standards were aliquoted to 500 µL-portions in 1.8-ml glass screw-cap vials.

A linear calibration curve was obtained by plotting the quotients of the peak areas of the analytes and labelled internal standards against the spiked concentration. For 3,5-DCC, D3-2,4-DCBA was used as internal standard, as both peaks nearly co-eluted from the column, providing good compensation of matrix effects for both analytes as shown in the validation of the method. The slope of these graphs was used to calculate the unknown concentrations of the analytes in urine samples. Unknown urine samples exceeding the upper calibration point were diluted with water and reprocessed as described. A reagent blank (consisting of processed water) as well as the unspiked pooled urine used for calibration was also included in every analytical series. Accuracy of the method was checked by spiking 6 different urine samples with variable creatinine content (0.46–2.96 g/L) with 30 µg/L of 3,5-DCC and 2,4-DCP as well as 6 µg/L for 3,5-DCP and was determined to be 110.4% (99.3–120.9%), 99.3% (93.6–105%) and 103.4% (101.3–105.2%), respectively. The LOQs for 3,5-dichlorocatechol and 2,4-dichlorophenol were determined to be 10 µg/L urine, while the sensitivity of 3,5-dichlorophenol was remarkably better with an LOQ of 0.2 µg/L urine. For instrument control and quantitative analysis, Analyst 1.6.3 and MultiQuant 2.1 were used (Sciex, Darmstadt, Germany). A chromatogram of the processed urine sample of a volunteer is shown in the Supplement (Figure S2).

### Subjective ratings of odour intensity and annoyance

Parallel to the exposure, subjective perception of the odour intensity and annoyance was assessed via an online survey created using a survey framework called “formr” (Arslan et al. 2020). Volunteers could access this online survey via their mobile phones while being in the exposure chamber. Subjective ratings of odour were collected before entering the room on all testing days for all volunteers and after 1, 2, 3, 4, 5, 15, 30, 60, and 120 min after entering the exposure chamber both in the morning and after the lunch break. Subjective perception was assessed with the use of two items reflecting intensity and affective evaluation of the smell: 1) “Please check the surrounding air carefully. How strongly do you currently perceive an odour?” and 2) “How annoying to you is the odour (smell) now?” Both items were rated on a 5-point scale ranging from 1 (“Not perceptible”) to 5 (“Extremely strongly”) for the first item and from 1 (“Not annoying”) to 5 (“Extremely annoying”) for the second item (VDI 3883–1, VDI 3883–2). Higher scores on both items indicated higher levels of odour intensity and annoyance.

### Environmental worry

Environmental worry is considered a moderator variable that can alter the perceived odour annoyance and is recommended to be included in the analysis. Volunteers’ environmental worry was assessed using a short version of the German scale “Umweltbesorgnis” (Hodapp and Schwarzer 1996). The short version, presented in VDI 3883–1, included 5 items, which were (1) “I often think about taking pollutants into my body”, (2) “I am worried that environmental toxins will affect my mental abilities”, (3) “Poor memory could also come from too many chemicals in our environment”, (4) “I fear my organism is already damaged by dangerous environmental substances” and (5) “When I travel, I consider beforehand where I will be least exposed to pollutants in the water or air”. Items were rated on a 4-point Likert scale ranging from 1 (“fully disagree”) to 4 (“fully agree”). These questions were administered only once at the end of the 3^rd^ participation day in order to avoid interference with volunteers’ reaction towards the environmental conditions in the exposure chamber. The responses to the five items are summed to obtain the degree of environmental worry. Higher scores indicate more thoughts on the effects that toxins and chemicals could have on someone’s physical and mental health. In addition, the number of “concerned persons” is the number of persons responding with “agree or “fully agree” on one or more of the five items (VDI 3883–1).

### Statistical analysis

The data analysis of metabolite levels was performed using Microsoft Excel 2010. The metabolite concentrations c(t) after reaching the maximum excretion (c_max_) follow an exponential decay according to the formula:

$$c\left( t \right)\, = \,c_{\max } \times e^{ - k\Delta t} ,$$

with *k* as a rate constant of urinary excretion and Δt as time after maximum concentration in hours. Then, the individual half-time of excretion (t_1/2_) was calculated as t_1/2_ = ln2/k.

The statistical analysis of the subjective ratings was conducted using the open-source software R Studio (version 3.6.3). Due to non-normally distributed data for perceived odour intensity and annoyance, Friedman’s tests were conducted to compare the effect of the exposure on subjective odour intensity and annoyance with Kendall’s W as effect size measure. Post hoc, pairwise comparisons using Wilcoxon rank sum test with Bonferroni correction were performed to explore differences between individual exposures.

Due to the small number of participants, environmental worry will be used to describe the population, but not added to the statistical analysis.

## Results and discussion

### Determination of 1,3-DCB in whole blood

**Fig. 4:**
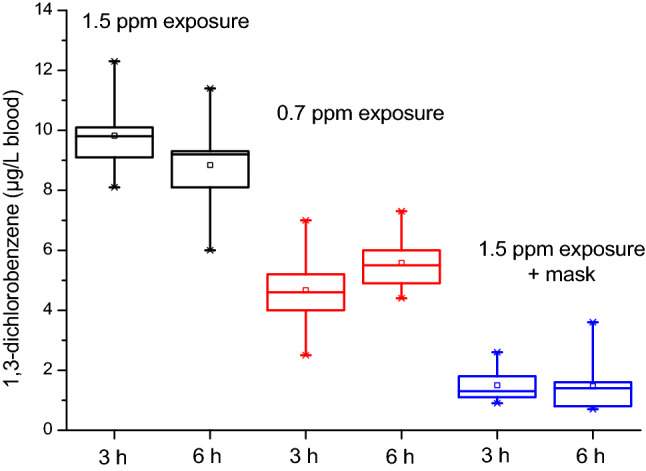
Concentrations of 1,3-dichlorobenzene determined in blood of the volunteers (*n* = 10) for the different exposure scenarios at the two time points

1,3-DCB was quantified in every blood sample taken after 3 and 6 h of exposure in the AWSL. The concentrations at the two time points (3 and 6 h) were not significantly different (two-sided *t* test, *p* > 0.05) with median values of 9.7 µg/L and 9.0 µg/L for 1.5 ppm exposure, 4.5 and 5.4 µg/L for 0.7 ppm exposure and 1.3 and 1.4 µg/L for 1.5 ppm exposure with face mask, respectively. This proves the rapid reaching of a steady state for 1,3-DCB in blood. The concentrations determined in the different exposure scenarios after 3 and 6 h are displayed in Fig. 4. As visible from Fig. 4, the face mask clearly reduces the internal exposure of the volunteers up to 84% as compared to the same exposure without face mask, underlining the efficacy of correct personal protective measures. Remarkably, one (of three) volunteers wearing a beard (volunteer 6) displayed the highest blood levels under this exposure scenario, which is plausibly explained with slight leakage in the mask due to beard hair.

### Determination of urinary metabolites of 1,3-DCB

Overall, we obtained between 3 and 15 urine samples from the volunteers over the 24-h collection period with total urine volumes ranging between 0.93 and 4.08 L. The creatinine content of the individual urine samples ranged from 0.12 g/L to 5.59 g/L. In order to provide the full dataset, the urine samples with creatinine values < 0.3 and > 3 g/L were not excluded from the evaluation (*n* = 38 of 203 urine samples did not meet these criteria). The metabolites 3,5-DCC, 2,4-DCP and 3,5-DCP were consistently detected above LOQ in every urine sample over the study period, except for the pre-exposure urine sample given before the first week’s exposure (1.5 ppm).

**Fig. 5:**
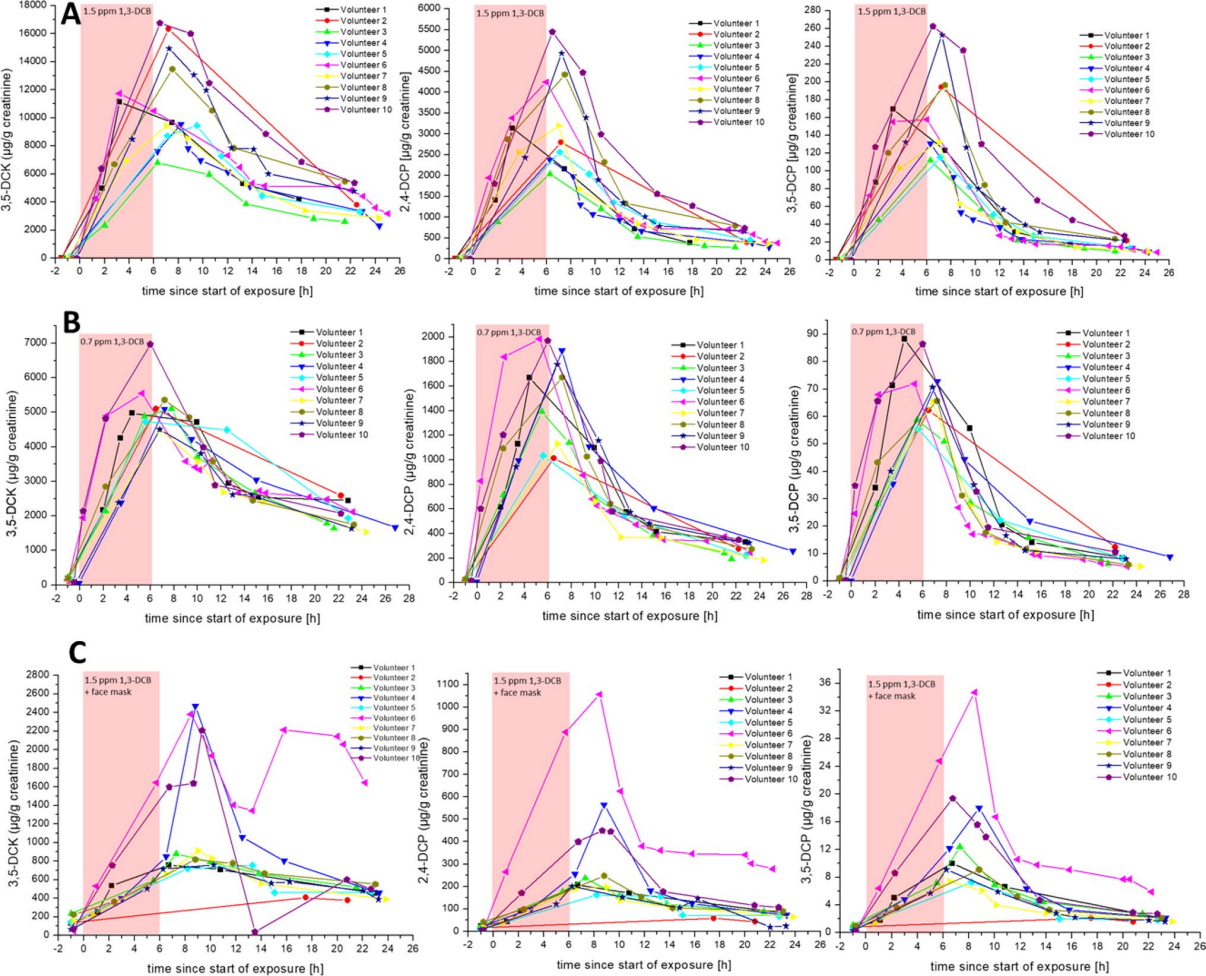
Time course of the creatinine-corrected urinary excretion of the metabolites 3,5-DCC (left), 2,4-DCP (middle) and 3,5-DCP (right) over 24 h after the start of exposure to 1,3-DCB at 1.5 ppm (**A**), 0.7 ppm (**B**) and 1.5 ppm + face mask (**C**)

To our surprise, even a time span of 1 week without exposure was not sufficient to lead to non-quantifiable levels in the urine sample of the following week (especially for 3,5-DCC), although these pre-exposure levels were low and nearly negligible compared to the levels in the following samples. The time course of the urinary excretion of the metabolites in the different exposure scenarios is shown in Fig. 5 on a creatinine-corrected basis and shows the expected concentration profile with maximum values reached short after the end of exposure and levels decreasing afterwards with the corresponding urinary half-life of the metabolites. The volume-based time course is depicted in the Supplement to this manuscript (Fig. S3). The pharmacokinetic parameters obtained from these data are summarised in Table 2. The full dataset of the study is also provided in the Supplement to this manuscript (Table S3).

**Table 2:** Pharmacokinetic parameters for the urinary metabolites 3,5-DCC, 2,4-DCP and 3,5-DCP for all human volunteers and exposure scenarios

Exposure	*T*_max_ [h]*			*C*_max_ [µg/g crea.]			T_½_ [h]*			Total excretion _(24 h)_ [µg]		
	3,5-DCC	2,4-DCP	3,5-DCP	3,5-DCC	2,4-DCP	3,5-DCP	3,5-DCK	2,4-DCP	3,5-DCP	3,5-DCC	2,4-DCP	3,5-DCP
1.5 ppm												
Volunteer 1	3.2	3.2	3.2	11,148	3138	169	9.6	4.6	4.1	7950	1481	81
Volunteer 2	7.2	7.2	7.2	16,338	2796	194	7.3	5.4	4.8	17,115	2654	178
Volunteer 3	6.3	6.3	6.3	6806	2036	112	10.2	5.1	4.3	10,343	2274	113
Volunteer 4	8.2	6.3	6.3	9538	2373	131	9.5	6.5	5.4	12,923	2806	138
Volunteer 5	9.5	7.1	7.1	9449	2563	115	9.0	6.2	5.0	13,564	3117	126
Volunteer 6	3.2	6.0	6.0	11,740	4246	158	13.3	7.5	5.9	9186	2586	91
Volunteer 7	7.0	7.0	7.0	9441	3195	133	9.5	5.7	4.7	12,406	2886	112
Volunteer 8	7.5	7.5	7.5	13,475	4421	197	11.2	6.1	5.0	15,244	3979	159
Volunteer 9	7.3	7.3	7.3	14,934	4934	253	8.6	5.1	4.4	13,960	3360	156
Volunteer 10	6.5	6.5	6.5	16,763	5449	263	9.0	5.4	4.5	16,835	4132	198
Mean	6.6 ± 1.9	6.4 ± 1.2	6.4 ± 1.2	11,963 ± 3149	3515 ± 1108	173 ± 51	9.7 ± 1.5	5.8 ± 0.8	4.8 ± 0.5	12,953 ± 2922	2928 ± 742	135 ± 36
0.7 ppm												
Volunteer 1	4.5	4.5	4.5	4976	1670	88	15.8	7.4	5.1	5286	1175	56
Volunteer 2	6.5	6.5	6.5	5092	1012	62	16.1	8.4	6.7	6308	1009	58
Volunteer 3	7.8	5.5	5.5	5099	1392	59	9.1	5.8	5.1	8580	1818	74
Volunteer 4	7.2	7.2	7.2	5095	1890	73	12.3	7.2	6.7	8453	2242	84
Volunteer 5	5.6	5.6	5.6	4729	1035	56	12.8	7.8	6.4	4980	891	44
Volunteer 6	5.3	5.3	5.3	5547	1984	72	15.8	7.4	5.5	5594	1400	45
Volunteer 7	6.8	6.8	6.8	4490	1132	65	11.6	7.1	5.1	8105	1642	81
Volunteer 8	7.2	7.2	7.2	5356	1672	66	9.6	6.6	5.1	6056	1584	56
Volunteer 9	6.8	6.8	6.8	4504	1777	71	11.0	6.6	5.3	4808	1479	51
Volunteer 10	6.0	6.0	6.0	6969	1970	86	10.2	7.0	5.9	5400	1318	54
Mean	6.4 ± 1.0	6.1 ± 0.9	6.1 ± 0.9	5186 ± 676	1553 ± 363	70 ± 10	12.4 ± 2.5	7.1 ± 0.7	5.7 ± 0.6	6357 ± 1394	1356 ± 377	60 ± 14
1.5 ppm + face mask												
Volunteer 1	6.7	6.7	6.7	758	208	10	21.7	12.2	7.4	829	186	7
Volunteer 2	17.5	17.5	17.5	405	59	2	31.5	7.3	9.1	489	65	2
Volunteer 3	7.3	7.3	7.3	880	137	12	18.7	10.7	6.9	2261	492	20
Volunteer 4	8.8	8.8	8.8	2469	565	18	6.4	5.3	4.9	1474	310	12
Volunteer 5	13.3	8.2	8.2	757	163	7	18.7	10.8	7.0	1987	402	15
Volunteer 6	8.5	8.5	8.5	2382	1057	35	5.5**	3.0**	2.6**	2226	788	22
Volunteer 7	9.0	9.0	6.5	911	198	7	12.4	10.3	8.6	1280	276	9
Volunteer 8	8.8	8.8	8.8	817	248	9	24.8	10.8	7.2	2245	513	17
Volunteer 9	10.3	6.3	6.3	759	201	9	15.8	4.9	6.8	1011	184	7
Volunteer 10	9.3	8.7	6.8	2207	449	19	7.1	6.6	5.5	1646	440	16
Mean	10.0 ± 3.0	9.0 ± 3.0	8.5 ± 3.1	1235 ± 746	329 ± 282	13 ± 9	16.3 ± 8.1	8.2 ± 3.0	6.6 ± 1.8	1545 ± 605	366 ± 197	13 ± 6

*calculated using creatinine-corrected values

**calculated omitting the last 4 urine samples with creatinine < 0.3 g/L

There is a clear dose–response in the excretion of the urinary metabolites. All urinary metabolites correlated excellently with each other, as reported previously in our report on workers in the silicone industry (Schettgen et al. 2022). As described previously, 3,5-DCC is by far the metabolite with the highest concentrations observed, followed by 2,4-DCP and – to a lesser extent – 3,5-DCP. 3,5-DCC also displayed the longest urinary half-life with mean values ranging between 9.7 and 16.3 h for the different exposure scenarios, pointing to a possible carry-over to the next shift in occupational studies. This is also in good agreement with data reported for 3,4- and 2,3-DCC as metabolites of the isomer 1,2-DCB (*t*_½_ = 8.3 ± 0.47 h) as summarised by the DFG Senate Commission (DFG 2010; Knecht and Jelke 1995).

There is clear evidence that the urinary half-life of all metabolites is increasing over the course of the study (see Table 2 and Figure S4). This could be explained with biphasic urinary elimination kinetics, although we had no hint for this from the kinetics over the 24-h urine collection period. It would also explain the surprising urinary metabolite levels in the first urine samples after 1 week without exposure to 1,3-DCB. Moreover, the lipophilic 1,3-DCB might be stored to a small extent in fatty tissues and re-distribute in the blood stream as shown in exposed rats (Kimura et al. 1984), leading to a low-level excretion of urinary metabolites long after the wash-out phase.

As visible from Fig. 5 and Table 2, the absorption of 1,3-DCB in the exposure scenario with the face mask is clearly delayed (*T*_max_ increased about 3 h for all metabolites compared to the inhalative exposure scenario), as it would be expected from a dermal absorption from the atmosphere. Moreover, also the urinary half-life of all metabolites is also highest under this exposure scenario, which has also been reported for dermal absorption of NMP from the atmosphere (Bader et al. 2008). Overall, the use of the face mask reduced the excretion of 1,3-DCB metabolites to about 90% compared to the exposure of 1.5 ppm, which is in good congruence with the results of the blood measurements of 1,3-DCB.

### Subjective ratings of odour intensity and annoyance

The volunteers reported an average odour intensity of 1.7 across all three conditions (*S.D.* = 0.7), indicating that the strength of the odour was almost not perceptible. In addition, the average level of annoyance reported was 1.3 across conditions (S.D. = 0.5), indicating minimal to no annoyance. In contrast to the results from the analysis of metabolites, no clear dose–response was observed neither for perceived odour intensity nor for perceived odour annoyance (see Table 3). The Friedman’s test to compare the effect of exposure on perceived odour intensity resulted in a significant difference between at least two groups (*Χ*^2^(2) = 15.8, *p* = 0.0004, *W* = 0.79 (large)). However, post hoc pairwise comparisons using Wilcoxon rank sum test with Bonferroni correction showed that perceived odour intensity differed significantly between 0.7 ppm and 1.5 ppm with face mask (*p*_adj_ = 0.002) and between 1.5 ppm and 1.5 ppm with face mask (*p*_adj_ = 0.0004), but not between 0.7 ppm and 1.5 ppm (*p*_adj_ = 0.30). The Friedman’s test to compare the effect of exposure on perceived odour annoyance also found a significant difference between at least two groups (*Χ*^2^ (2) = 7.6, *p* = 0.02, *W* = 0.38 (moderate). Nevertheless, perceived odour annoyance solely differed between 1.5 ppm and 1.5 ppm with face mask (*p*_adj_ = 0.041), but not between the other two conditions (0.7 ppm vs. 1.5 ppm with face mask: *p*_adj_ = 0.2; 0.7 ppm vs. 1.5 ppm: *p*_adj_ = 1). Interestingly, the above reported potential leakage in the mask due to beard hair (volunteer 6) is observable also in the subjective data and in volunteer 10, who showed also higher metabolite levels (probably also due to mask leakage). In exposure 1.5 ppm with face mask, votings are significantly higher for these participants compared to others.

**Table 3:** Subjective ratings of odour intensity and annoyance as determined in the 5-point scale of the questionnaire

		Intensity	Annoyance
Exposure	*n*	mean ± SD	mean ± SD
1.5 ppm	10	2.2 ± 0.3	1.5 ± 0.5
0.7 ppm	10	1.9 ± 0.3	1.3 ± 0.5
1.5 ppm + face mask	10	1.1 ± 0.3	1.0 ± 0.0

**Fig. 6:**
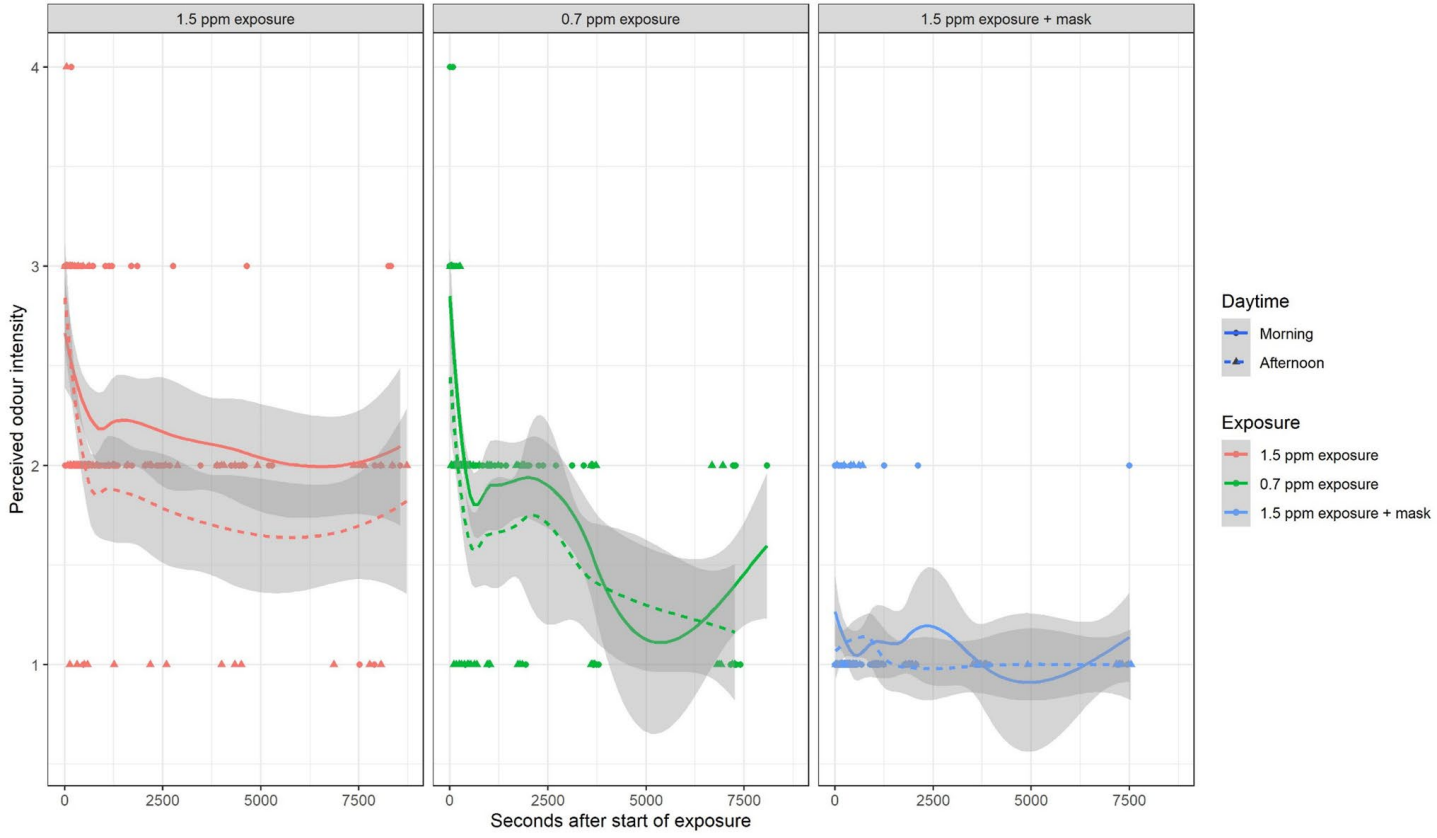
Time course of perceived odour intensity by exposure and daytime. Lines show Loess regression fits with standard error ranges in grey

**Fig. 7:**
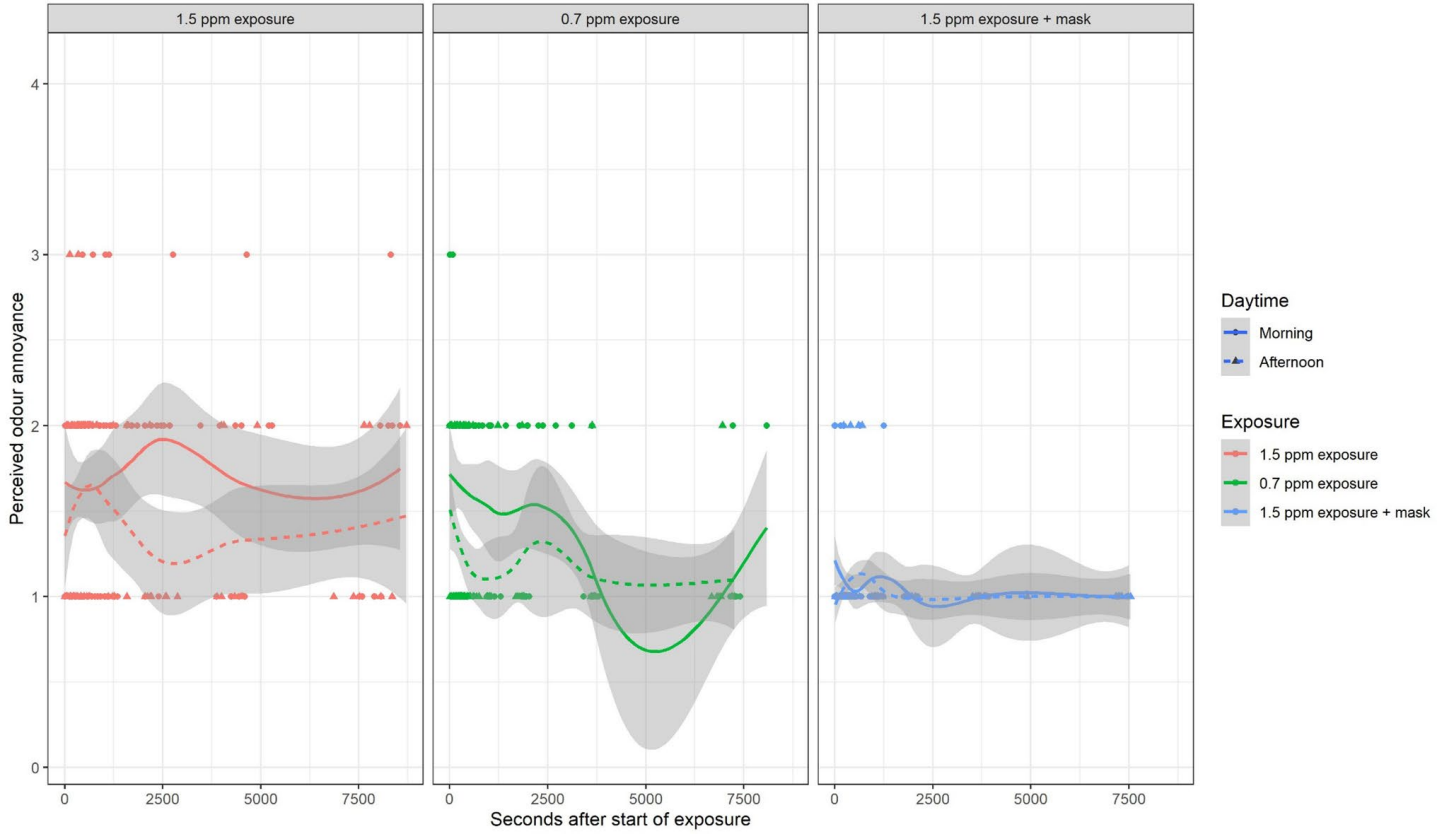
Time course of perceived odour annoyance by exposure and daytime. Lines show Loess regression fits with standard error ranges in grey

As to be expected based on studies of olfactory adaptation to other odours (Cometto-Muñiz and Cain 1995), the odour intensity dropped significantly within few minutes after the start of exposure also, when exposed to 0.7 ppm 1,3-DCB, while the odour intensity reported by volunteers when exposed to 1.5 ppm 1,3-DCB vapour dropped only slightly and remained at steady levels. In the afternoon, i.e. after having spent the lunch break outside the exposure, similar patterns are observed, while the steady level during 1.5 ppm 1,3 DCB is lower than in the morning (see Fig. 6). The time course for odour annoyance does not show any similar trends as ratings are in general low, i.e. volunteers reported hardly any perceived odour annoyance from the beginning of each exposure (see Fig. 7).

Overall, the group of volunteers can be characterised with a just below average level of environmental worry (mean 11.3 ± 2.71), which is lower, but comparable to study populations in earlier studies (Steinheider and Hodapp, 1998). At the same time, 9 out of 10 volunteers are grouped as concerned.

### Strengths and limitations

Our study has clear strengths and limitations. A clear strength is the validity and stability of the exposure conditions in the AWSL with online measurements of 1,3-DCB using the Gasmet analyser (see Fig. 3). Also the methods of biological monitoring (blood and urine measurements) were performed using validated, quality controlled and highly specific methods with state-of-the-art mass spectrometry, including isotopically labelled internal standards for the metabolites (except 3,5-DCC).

A clear limitation is the lack of physical exercise of the human volunteers in the AWSL. Thus, our exposure conditions are not fully representative for the typical workload of workers during shift and might be a “best-case scenario” with a low respiratory volume. Furthermore, although our data give a good (and realistic) hint on the contribution of dermal absorption on internal exposure to 1,3-DCB, the use of appropriate face masks does not exclude potential leakages that might have distorted our results. The use of air-ventilated helmets for the volunteers was not feasible in the AWSL for technical reasons. Another limitation is the lack of data on temperature and humidity in the AWSL during the experiments, as these parameters were shown to strongly affect percutaneous absorption of solvent vapours (Mraz and Nohova 1992). Nevertheless, our results point to a contributional uptake of 1,3-DCB vapours from the atmosphere as previously shown for other solvents, like NMP (Mraz and Nohova 1992; Bader et al. 2008). A minor limitation of our study might be that we missed to determine the urinary mercapturic acids of 1,3-DCB that were reported to be the main metabolites in rat experiments (Kimura et al. 1984, 1992) and are also relevant in humans (Zenser et al. 1997). With respect to the subjective ratings, the sample size is too small for generalizable conclusions due to large inter- and intrapersonal variances in human perception. Future studies need to work with larger samples.

## Conclusion, proposal of a biological limit value and need for psychological limit value

With this study we provide important data on the quantitative relationship between inhalative exposure to the solvent 1,3-DCB and excretion of the specific metabolites 3,5-DCC, 2,4-DCP and 3,5-DCP in humans. To our knowledge, this is the first study worldwide to provide such data that set the basis for the evaluation of a biological limit value for 1,3-DCB. As 1,3-DCB is formed in the production of silicone rubber as decomposition product of bis(2,4-dichlorobenzoyl)peroxide (2,4-DCBP) with many workers exposed worldwide, a biological limit value is highly warranted.

The correlation between air levels and blood and urine measurement concentrations (at T_max_) gives the following equations:

1. 1,3-DCB (blood, µg/L) = 5.26 × 1,3-DCB (air, ppm) + 1.45,
2. 3,5-DCC (urine, µg/g crea.) = 8472 × 1,3-DCB (air, ppm) –744,
3. 2,4-DCP (urine, µg/g crea.) = 2452 × 1,3-DCB (air, ppm) –163,
4. 3,5-DCP (urine, µg/g crea.) = 128.4 × 1,3-DCB (air, ppm) –20.

Under consideration of the time the volunteers were exposed to 1,3-DCB (6-h vs. 8-h shift); this would correspond to the following proposed biological limit values, corresponding to an exposure to 1,3-DCB for 8 h at the current threshold limit value of 2 ppm: 1,3-DCB (B) 12 µg/L blood; 3,5-DCC (U) 21.600 µg/g crea.; 2,4-DCP (U) 6.300 µg/g crea and 3,5-DCP (U) 320 µg/g crea. All these values are based on the mean values of the volunteers under the described exposures, as proposed by the Deutsche Forschungsgemeinschaft for the derivation of biological limit values (BAT values) (DFG 2022). From our data, the recommended sampling time would be the end of shift; however, the high urinary half-life of 3,5-DCC might lead to a carry-over from previous shifts. The values derived in our study are slightly lower compared to the biological limit value for the structural isomer 1,4-DCB (threshold limit value: 2 ppm), which was set to be 10 mg/l urine for the metabolite 2,5-DCP (DFG 2022). However, this biological limit value was evaluated on the basis of an early study with photometric quantification of urinary 2,5-DCP (Pagnotto and Walkley 1965).

Limits due to the perceived annoyance of the odour do not exist. While the odour was perceived significantly different with and without mask, but not with increasing exposure intensity, annoyance ratings were in general very low. Human odour adaptation leads to a lowering of perceived intensity, so that in this case, neither perception nor annoyance are meaningful indicators to describe potential psychological burden. At the same time, this laboratory exposure in these three experiments cannot give suggestions related to limits due to perceived annoyance for the potential burden of long-term real-life exposures.

## Supplementary Information

Below is the link to the electronic supplementary material.

Supplementary file1 (DOCX 3049 KB)

## Data Availability

The datasets generated during and/or analysed during the current study are available from the corresponding author on reasonable request (if not contained in the Supplemental Material).
