# Supplementary material for: Excretion kinetics of 1,3-dichlorobenzene and its urinary metabolites after controlled airborne exposure in human volunteers
Source: Arch Toxicol. 2023 Jan 30;97(4):1033–45. doi: 10.1007/s00204-023-03447-x (PMC10025240; doi:10.1007/s00204-023-03447-x)
Supplement: Supplementary file 1 — Supplementary file1 (DOCX 3049 KB) [file 204_2023_3447_MOESM1_ESM.docx]

Figure S1: Picture of the 1,3-dichlorobenzene vapor generator.


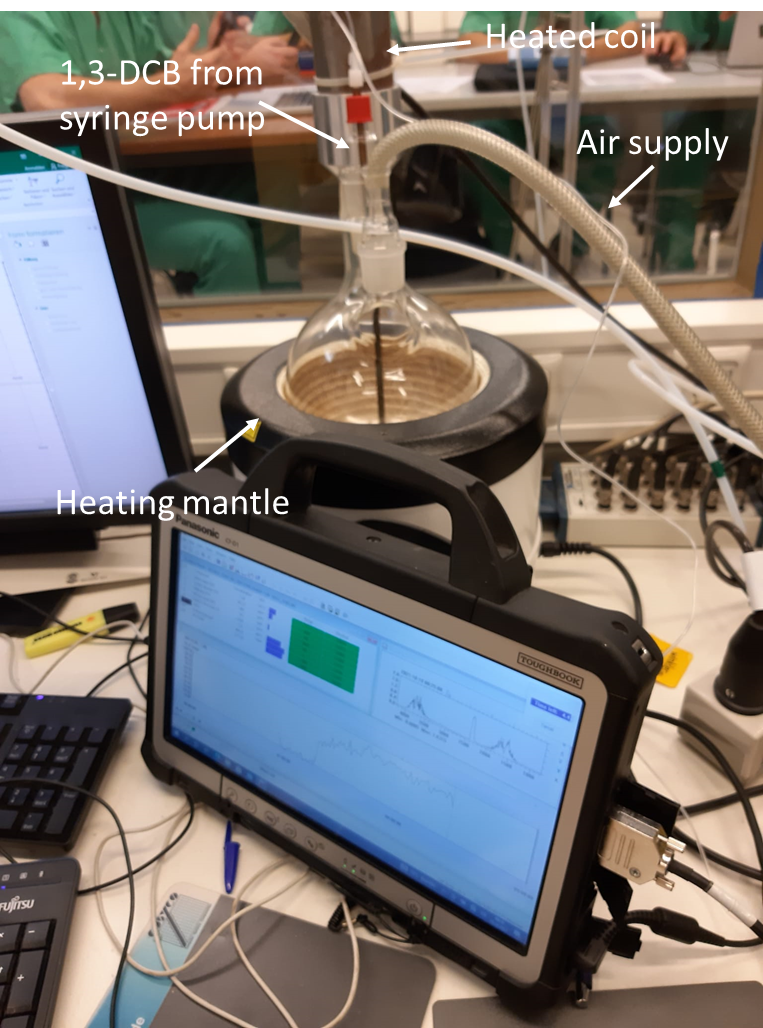


Table S1: Analysis program of the HPLC pumps and switching valve.

|  |  | **Pump 1** | |  | **Pump 2** | |  |  |
| --- | --- | --- | --- | --- | --- | --- | --- | --- |
| **Time** | **Valve Pos.** | **Eluent A**  **(%)** | **Eluent B**  **(%)** | **Flow-rate**  **(ml/min)** | **Eluent A**  **(%)** | **Eluent B**  **(%)** | **Flow-rate**  **(ml/min)** | **Analysis step** |
| 0 | A | 88 | 12 | 0.95 | 50 | 50 | 0.4 | *clean-up* |
| 5 | B | 88 | 12 | 0.95 |  |  |  | *Transfer* |
| 6.5 | B | 88 | 12 | 0.3 |  |  |  |  |
| 7 | A | 88 | 12 | 0.3 |  |  |  |  |
| 8.5 | A | 88 | 12 | 0.3 | 50 | 50 | 0.4 |  |
| 10 | A | 0 | 100 | 0.3 |  |  |  |  |
| 11.5 | A |  |  |  | 18 | 88 | 0.4 | *Separation and washing* |
| 13.5 | A |  |  |  | 0 | 100 | 0.4 |  |
| 18 | A |  |  |  | 0 | 100 | 0.4 |  |
| 21 | A |  |  |  | 50 | 50 | 0.4 |  |
| 22 | A | 0 | 100 | 0.3 |  |  |  |  |
| 23 | A | 88 | 12 | 0.3 |  |  |  | *Reconditioning* |
| 24 | A | 88 | 12 | 0.95 |  |  |  |  |
| 25 | A | 88 | 12 | 0.95 | 50 | 50 | 0.4 |  |

Table S2: Retention times and selected MRM-parameters for the analytes and internal standards. DP: declustering potential (V); CE: collision energy (V); CXP: collision exit potential (V)

| **Analyte** | **Retention time**  **(min)** | **Parent ion**  **(Q 1)** | **Daughter ion**  **(Q 3)** | **DP** | **CE** | **CXP** |  |
| --- | --- | --- | --- | --- | --- | --- | --- |
| D_3_-2,4-DCBA | 10.91 | 193.9 | 149.9 | -45 | -12 | -11 |  |
| 3,5-DCC | 10.70 | 178.8 | 105.0 | -60 | -26 | -7 | *Quantifier* |
|  |  | 176.8 | 105.0 | -60 | -26 | -7 | *Qualifier* |
| 2,4-DCP | 11.46 | 160.9 | 124.9 | -85 | -24 | -9 | *Quantifier* |
|  |  | 162.9 | 124.9 | -85 | -24 | -9 | *Qualifier* |
| D_3_-2,4-DCP | 11.45 | 165.9 | 128.9 | -85 | -24 | -9 |  |
| 3,5-DCP | 11.84 | 160.9 | 124.9 | -85 | -24 | -9 | *Quantifier* |
|  |  | 162.9 | 124.9 | -85 | -24 | -9 | *Qualifier* |
| D_3_-3,5-DCP | 11.83 | 165.9 | 128.9 | -85 | -24 | -9 |  |

Figure S2: Section of the chromatogram of a processed urine sample of volunteer 3 (36 yrs, m) with a creatinine content of 1.6 g/L.


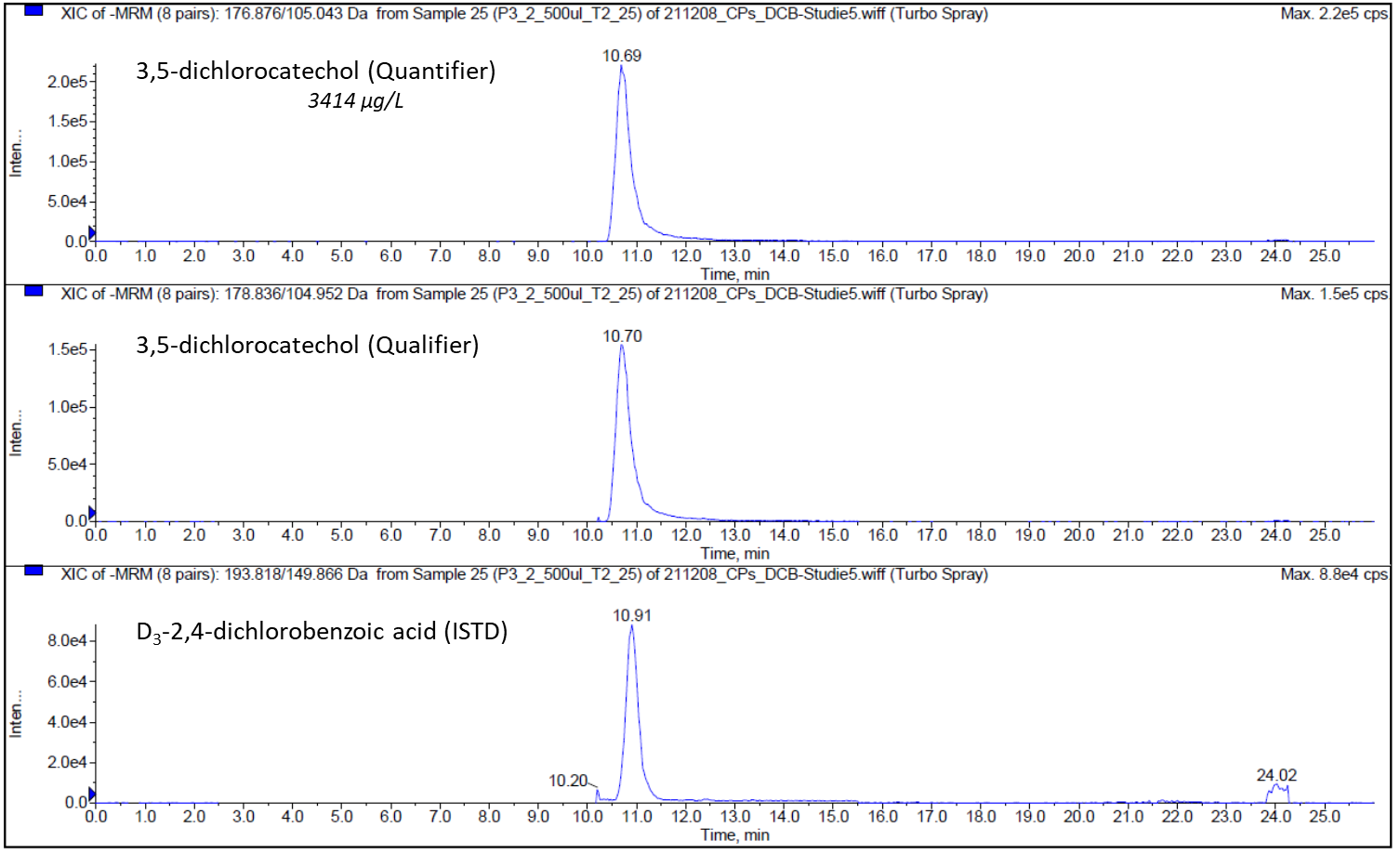


Figure S2 (ctd.): Section of the chromatogram of a processed urine sample of volunteer 3 (36 yrs, m) with a creatinine content of 1.6 g/L.


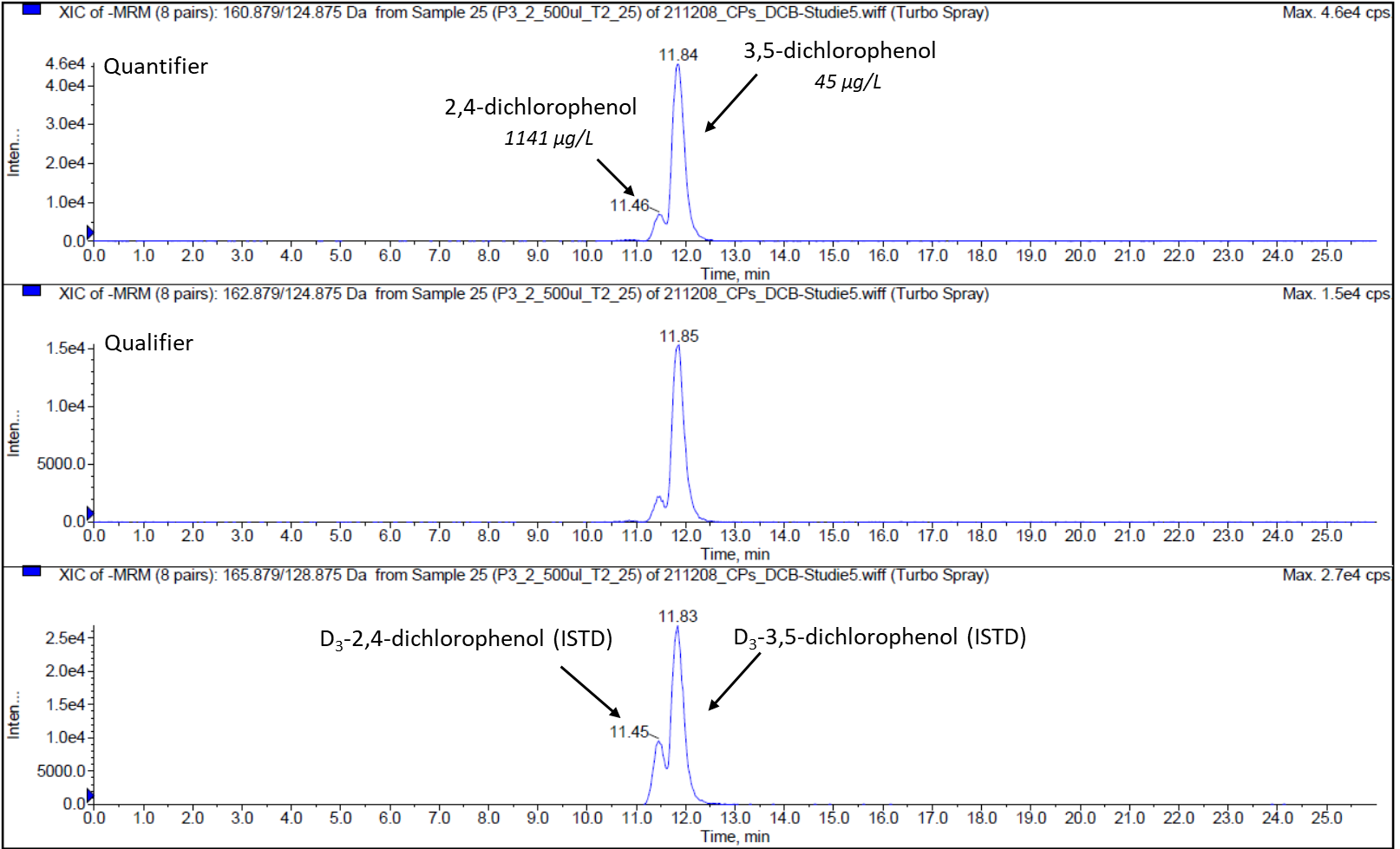


Figure S3: Time course of the volume-based urinary excretion of the metabolites 3,5-DCC (left), 2,4-DCP (middle) and 3,5-DCP (right) over 24 h after the start of exposure to 1,3-DCB at 1.5 ppm (A), 0.7 ppm (B) and 1.5 ppm + face mask (C).


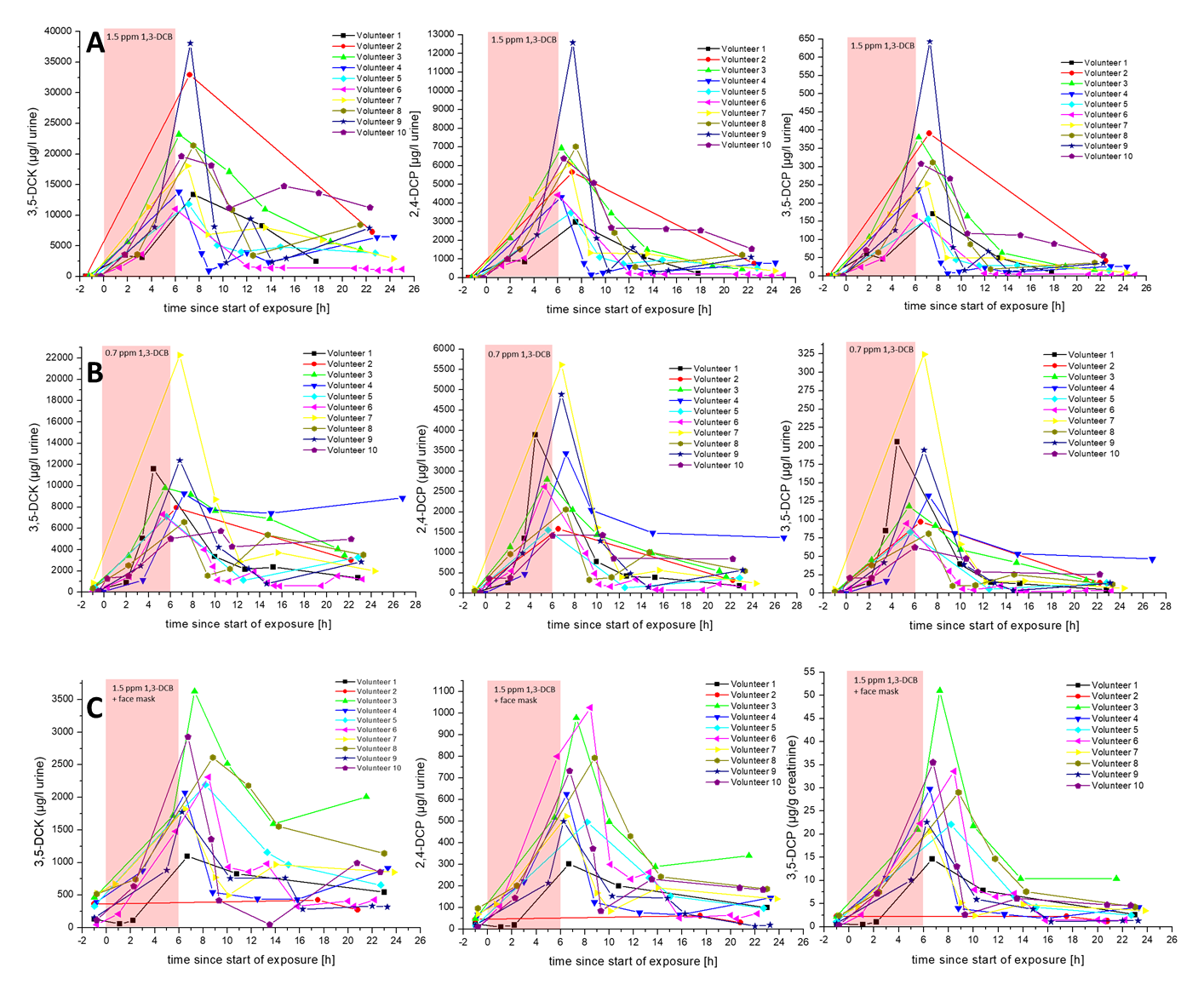


Figure S4: Urinary half-lives of the metabolites determined in the course of the study.


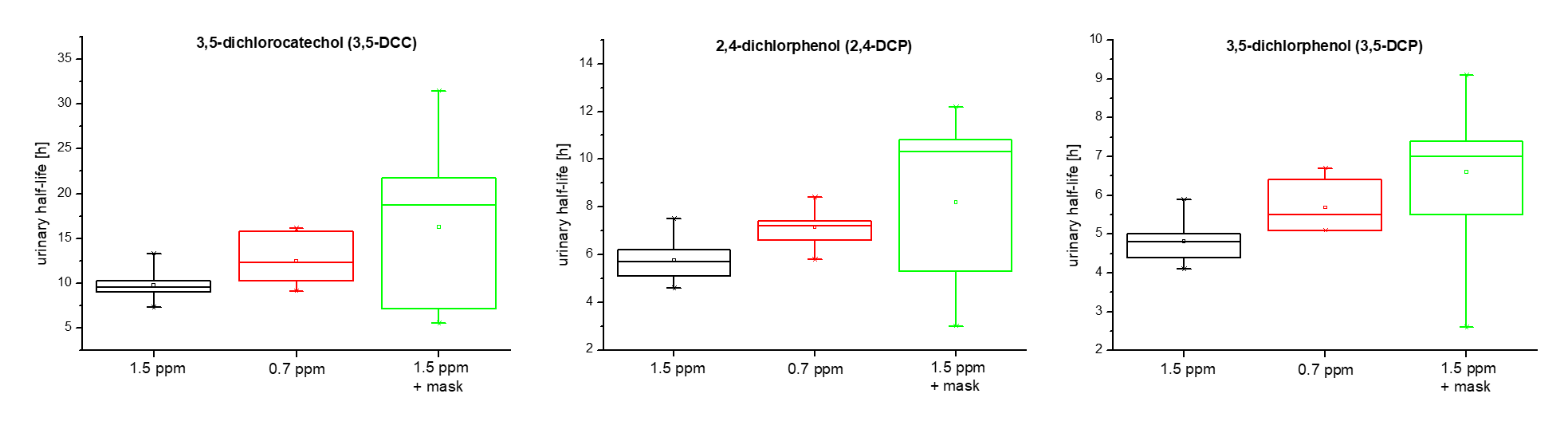


Table S3: Full data on excretion of urinary metabolites of 1,3-DCB in all volunteers.

| Volunteer 1 | | | | | | | | | | | | | | |  |
| --- | --- | --- | --- | --- | --- | --- | --- | --- | --- | --- | --- | --- | --- | --- | --- |
| 1.5 ppm 1,3-DCB | | | | | 0.7 ppm 1,3-DCB | | | | | 1.5 ppm 1,3-DCB + face mask | | | | |  |
| time  [h] | crea.  [g/L] | 3,5-DCC  [µg/L] | 2,4-DCP  [µg/L] | 3,5-DCP  [µg/L] | time  [h] | crea.  [g/L] | 3,5-DCC  [µg/L] | 2,4-DCP  [µg/L] | 3,5-DCP  [µg/L | time  [h] | crea.  [g/L] | 3,5-DCC  [µg/L] | 2,4-DCP  [µg/L] | 3,5-DCP  [µg/L | |
| - 1.5 | 0.79 | < 10 | < 10 | < 0.1 | - 1 | 0.60 | 112 | 14 | 0.7 | - 1 | 1.03 | 133 | 24 | 0.7 | |
| 1.8 | 0.69 | 3450 | 976 | 60.4 | 2.0 | 0.41 | 888 | 252 | 13.9 | 1.1 | *0.23* | *58* | *10* | *0.4* | |
| 3.2 | *0.27* | *3036* | *855* | *46.1* | 3.45 | 1.19 | 5067 | 1345 | 84.8 | 2.2 | *0.21* | *112* | *19* | *1.0* | |
| 7.5 | 1.38 | 13362 | 2982 | 170 | 4.45 | 2.33 | 11593 | 3890 | 206 | 6.7 | 1.45 | 1099 | 301 | 14.6 | |
| 13.25 | 1.55 | 8287 | 1130 | 49.0 | 9.95 | 0.71 | 3346 | 780 | 39.6 | 10.8 | 1.17 | 827 | 200 | 7.8 | |
| 17.8 | 0.59 | 2474 | 226 | 10.1 | 12.66 | 0.73 | 2150 | 419 | 15.0 | 23.0 | 1.20 | 545 | 99 | 2.6 | |
|  |  |  |  |  | 15.2 | 0.93 | 2360 | 386 | 13.1 |  |  |  |  |  | |
|  |  |  |  |  | 22.8 | 0.55 | 1345 | 182 | 4.7 |  |  |  |  |  | |
|  |  |  |  |  |  |  |  |  |  |  |  |  |  |  | |
| 1,3-DCB (B) 3 h: 9.1 µg/L  1,3-DCB (B) 6 h: 7.6 µg/L  Total excreted volume: 1602 mL  Total excreted dose  3,5-DCK: 7950 µg  2,4-DCP: 1481 µg  3,5-DCP: 81 µg | | | | | 1,3-DCB (B) 3 h: 5.0 µg/L  1,3-DCB (B) 6 h: 4.4 µg/L  Total excreted volume: 2416 mL  Total excreted dose  3,5-DCK: 5286 µg  2,4-DCP: 1175 µg  3,5-DCP: 56 µg | | | | | 1,3-DCB (B) 3 h: 1.1 µg/L  1,3-DCB (B) 6 h: 0.7 µg/L  Total excreted volume: 2068 mL  Total excreted dose  3,5-DCK: 829 µg  2,4-DCP: 186 µg  3,5-DCP: 7 µg | | | | |  |

| Volunteer 2 | | | | | | | | | | | | | | |
| --- | --- | --- | --- | --- | --- | --- | --- | --- | --- | --- | --- | --- | --- | --- |
| 1.5 ppm 1,3-DCB | | | | | 0.7 ppm 1,3-DCB | | | | | 1.5 ppm 1,3-DCB + face mask | | | | |
| time  [h] | crea.  [g/L] | 3,5-DCC  [µg/L] | 2,4-DCP  [µg/L] | 3,5-DCP  [µg/L] | time  [h] | crea.  [g/L] | 3,5-DCC  [µg/L] | 2,4-DCP  [µg/L] | 3,5-DCP  [µg/L | time  [h] | crea.  [g/L] | 3,5-DCC  [µg/L] | 2,4-DCP  [µg/L] | 3,5-DCP  [µg/L |
| - 1.5 | 2.25 | < 10 | < 10 | < 0.1 | - 1 | 2.31 | 290 | 32 | 1.9 | - 1 | 2.70 | 365 | 46 | 2.2 |
| 7.2 | 2.02 | 32900 | 5640 | 391 | 6.5 | 1.56 | 7944 | 1579 | 97.0 | 17.5 | 1.05 | 426 | 62 | 2.2 |
| 22.5 | 1.91 | 7267 | 758 | 41 | 22.2 | 1.16 | 3002 | 317 | 14.3 | 20.8 | 0.73 | 275 | 31 | 1.2 |
|  |  |  |  |  |  |  |  |  |  |  |  |  |  |  |
| 1,3-DCB (B) 3 h: 12.3 µg/L  1,3-DCB (B) 6 h: 8.8 µg/L  Total excreted volume: 1046 mL  Total excreted dose  3,5-DCK: 17115 µg  2,4-DCP: 2654 µg  3,5-DCP: 178 µg | | | | | 1,3-DCB (B) 3 h: 5.2 µg/L  1,3-DCB (B) 6 h: 5.3 µg/L  Total excreted volume: 1373 mL  Total excreted dose  3,5-DCK: 6308 µg  2,4-DCP: 1309 µg  3,5-DCP: 58 µg | | | | | 1,3-DCB (B) 3 h: 1.2 µg/L  1,3-DCB (B) 6 h: 1.1 µg/L  Total excreted volume: 1400 mL  Total excreted dose  3,5-DCK: 489 µg  2,4-DCP: 65 µg  3,5-DCP: 2 µg | | | | |

| Volunteer 3 | | | | | | | | | | | | | | |
| --- | --- | --- | --- | --- | --- | --- | --- | --- | --- | --- | --- | --- | --- | --- |
| 1.5 ppm 1,3-DCB | | | | | 0.7 ppm 1,3-DCB | | | | | 1.5 ppm 1,3-DCB + face mask | | | | |
| time  [h] | crea.  [g/L] | 3,5-DCC  [µg/L] | 2,4-DCP  [µg/L] | 3,5-DCP  [µg/L] | time  [h] | crea.  [g/L] | 3,5-DCC  [µg/L] | 2,4-DCP  [µg/L] | 3,5-DCP  [µg/L | time  [h] | crea.  [g/L] | 3,5-DCC  [µg/L] | 2,4-DCP  [µg/L] | 3,5-DCP  [µg/L |
| - 1.25 | 2.14 | < 10 | < 10 | < 0.1 | - 1 | 1.88 | 435 | 54 | 1.9 | - 1 | 1.97 | 463 | 50 | 2.2 |
| 2.0 | 2.39 | 5612 | 2114 | 107 | 2.2 | 1.60 | 3414 | 1141 | 45.0 | 5.5 | 2.91 | 1720 | 517 | 21.0 |
| 6.3 | *3.41* | *23202* | *6942* | *381* | 5.5 | 2.01 | 9816 | 2798 | 118 | 7.3 | *4.12* | *3627* | *978* | *51.0* |
| 10.5 | 2.87 | 17111 | 3453 | 164 | 7.8 | 1.80 | 9178 | 2053 | 91.6 | 10.8 | *3.17* | *2518* | *497* | *21.8* |
| 13.5 | 2.84 | 10974 | 1506 | 63.8 | 10.0 | 2.07 | 7665 | 1440 | 59.1 | 13.8 | 2.39 | 1594 | 290 | 10.4 |
| 19.0 | 2.00 | 5666 | 625 | 25.8 | 14.9 | 2.60 | 6904 | 991 | 41.6 | 21.5 | *3.86* | *2008* | *340* | *10.4* |
| 21.5 | 1.69 | 4405 | 465 | 16.7 | 21.0 | 2.28 | 4074 | 548 | 17.8 |  |  |  |  |  |
|  |  |  |  |  | 21.6 | 2.11 | 3463 | 404 | 13.3 |  |  |  |  |  |
|  |  |  |  |  |  |  |  |  |  |  |  |  |  |  |
| 1,3-DCB (B) 3 h: 9.2 µg/L  1,3-DCB (B) 6 h: 9.2 µg/L  Total excreted volume: 1011 mL  Total excreted dose  3,5-DCK: 10343 µg  2,4-DCP: 2274 µg  3,5-DCP: 113 µg | | | | | 1,3-DCB (B) 3 h: 4.3 µg/L  1,3-DCB (B) 6 h: 6.0 µg/L  Total excreted volume: 934 mL  Total excreted dose  3,5-DCK: 8580 µg  2,4-DCP: 1818 µg  3,5-DCP: 74 µg | | | | | 1,3-DCB (B) 3 h: 1.4 µg/L  1,3-DCB (B) 6 h: 1.9 µg/L  Total excreted volume: 1130 mL  Total excreted dose  3,5-DCK: 2261 µg  2,4-DCP: 492 µg  3,5-DCP: 20 µg | | | | |

| Volunteer 4 | | | | | | | | | | | | | | |
| --- | --- | --- | --- | --- | --- | --- | --- | --- | --- | --- | --- | --- | --- | --- |
| 1.5 ppm 1,3-DCB | | | | | 0.7 ppm 1,3-DCB | | | | | 1.5 ppm 1,3-DCB + face mask | | | | |
| time  [h] | crea.  [g/L] | 3,5-DCC  [µg/L] | 2,4-DCP  [µg/L] | 3,5-DCP  [µg/L] | time  [h] | crea.  [g/L] | 3,5-DCC  [µg/L] | 2,4-DCP  [µg/L] | 3,5-DCP  [µg/L | time  [h] | crea.  [g/L] | 3,5-DCC  [µg/L] | 2,4-DCP  [µg/L] | 3,5-DCP  [µg/L |
| - 1.0 | *3.64* | *< 10* | *< 10* | *< 0.1* | - 0.05 | 0.87 | 51 | < 10 | < 0.1 | - 0.8 | 2.60 | 394 | 66 | 2.0 |
| 6.3 | 1.82 | 13837 | 4313 | 237 | 3.5 | 0.47 | 1118 | 467 | 16.7 | 3.0 | 2.20 | 876 | 220 | 10.5 |
| 8.2 | 0.39 | 3753 | 780 | 36.5 | 7.2 | 1.82 | 9262 | 3436 | 132 | 6.5 | 2.44 | 2068 | 625 | 29.8 |
| 8.8 | *0.12* | *925* | *154* | *6.3* | 9.5 | 1.84 | 7762 | 2041 | 81.6 | 8.8 | *0.22* | *543* | *124* | *4.0* |
| 9.8 | *0.26* | *1817* | *279* | *11.9* | 15.0 | 2.44 | 7427 | 1479 | 53.5 | 12.5 | 0.42 | 444 | 77 | 2.7 |
| 12.0 | 0.64 | 3903 | 593 | 23.2 | 26.8 | *5.29* | *8851* | *1362* | *46.4* | 15.8 | 0.54 | 433 | 67 | 1.8 |
| 13.8 | 0.46 | 2343 | 305 | 10.6 |  |  |  |  |  | 23.3 | 2.00 | 915 | 145 | 4.2 |
| 22.9 | 1.91 | 6404 | 738 | 26.1 |  |  |  |  |  |  |  |  |  |  |
| 24.3 | 2.81 | 6484 | 798 | 25.4 |  |  |  |  |  |  |  |  |  |  |
|  |  |  |  |  |  |  |  |  |  |  |  |  |  |  |
| 1,3-DCB (B) 3 h: 8.1 µg/L  1,3-DCB (B) 6 h: 8.1 µg/L  Total excreted volume: 2874 mL  Total excreted dose  3,5-DCK: 12923 µg  2,4-DCP: 2806 µg  3,5-DCP: 138 µg | | | | | 1,3-DCB (B) 3 h: 3.2 µg/L  1,3-DCB (B) 6 h: 5.5 µg/L  Total excreted volume: 1614 mL  Total excreted dose  3,5-DCK: 8453 µg  2,4-DCP: 2242 µg  3,5-DCP: 84 µg | | | | | 1,3-DCB (B) 3 h: 0.9 µg/L  1,3-DCB (B) 6 h: 1.4 µg/L  Total excreted volume: 2270 mL  Total excreted dose  3,5-DCK: 1474 µg  2,4-DCP: 310 µg  3,5-DCP: 12 µg | | | | |

| Volunteer 5 | | | | | | | | | | | | | | |
| --- | --- | --- | --- | --- | --- | --- | --- | --- | --- | --- | --- | --- | --- | --- |
| 1.5 ppm 1,3-DCB | | | | | 0.7 ppm 1,3-DCB | | | | | 1.5 ppm 1,3-DCB + face mask | | | | |
| time  [h] | crea.  [g/L] | 3,5-DCC  [µg/L] | 2,4-DCP  [µg/L] | 3,5-DCP  [µg/L] | time  [h] | crea.  [g/L] | 3,5-DCC  [µg/L] | 2,4-DCP  [µg/L] | 3,5-DCP  [µg/L | time  [h] | crea.  [g/L] | 3,5-DCC  [µg/L] | 2,4-DCP  [µg/L] | 3,5-DCP  [µg/L |
| - 0.75 | 1.10 | < 10 | < 10 | < 0.1 | - 0.8 | 2.34 | 421 | 59 | 1.4 | - 1 | 2.32 | 332 | 17 | 1.1 |
| 7.1 | 1.36 | 11829 | 3479 | 156 | 5.6 | 1.50 | 7093 | 1552 | 83.2 | 8.2 | *3.05* | *2192* | *496* | *22.1* |
| 9.5 | 0.54 | 5106 | 1101 | 44.4 | 12.5 | *0.25* | *1122* | *137* | *5.6* | 13.3 | 1.53 | 1158 | 239 | 6.6 |
| 11.5 | 0.55 | 4003 | 747 | 28.1 | 22.8 | 1.69 | 3267 | 375 | 14.4 | 15.1 | 2.10 | 970 | 153 | 4.2 |
| 14.8 | 1.08 | 4817 | 968 | 29.3 |  |  |  |  |  | 22.7 | 1.42 | 655 | 97 | 2.5 |
| 22.8 | 1.16 | 3823 | 526 | 15.5 |  |  |  |  |  |  |  |  |  |  |
|  |  |  |  |  |  |  |  |  |  |  |  |  |  |  |
| 1,3-DCB (B) 3 h: 9.8 µg/L  1,3-DCB (B) 6 h: 9.3 µg/L  Total excreted volume: 2374 mL  Total excreted dose  3,5-DCK: 13564 µg  2,4-DCP: 3117 µg  3,5-DCP: 126 µg | | | | | 1,3-DCB (B) 3 h: 4.0 µg/L  1,3-DCB (B) 6 h: 5.0 µg/L  Total excreted volume: 1312 mL  Total excreted dose  3,5-DCK: 4980 µg  2,4-DCP: 891 µg  3,5-DCP: 44 µg | | | | | 1,3-DCB (B) 3 h: 1.8 µg/L  1,3-DCB (B) 6 h: 1.4 µg/L  Total excreted volume: 1593 mL  Total excreted dose  3,5-DCK: 1987 µg  2,4-DCP: 402 µg  3,5-DCP: 15 µg | | | | |

| Volunteer 6 | | | | | | | | | | | | | | |
| --- | --- | --- | --- | --- | --- | --- | --- | --- | --- | --- | --- | --- | --- | --- |
| 1.5 ppm 1,3-DCB | | | | | 0.7 ppm 1,3-DCB | | | | | 1.5 ppm 1,3-DCB + face mask | | | | |
| time  [h] | crea.  [g/L] | 3,5-DCC  [µg/L] | 2,4-DCP  [µg/L] | 3,5-DCP  [µg/L] | time  [h] | crea.  [g/L] | 3,5-DCC  [µg/L] | 2,4-DCP  [µg/L] | 3,5-DCP  [µg/L | time  [h] | crea.  [g/L] | 3,5-DCC  [µg/L] | 2,4-DCP  [µg/L] | 3,5-DCP  [µg/L |
| - 0.75 | 0.68 | < 10 | < 10 | < 0.1 | - 1 | 0.49 | 70 | < 10 | 0.2 | - 0.8 | 0.97 | 48 | < 10 | 0.2 |
| 1.3 | 0.34 | 1434 | 156 | 24.4 | 0.3 | *0.26* | *506* | *215* | *6.4* | 1.0 | 0.40 | 212 | 106 | 2.6 |
| 3.2 | 0.31 | 3649 | 1051 | 48.2 | 2.3 | 0.30 | 1470 | 552 | 20.4 | 5.75 | 0.90 | 1480 | 800 | 22.3 |
| 6.0 | 1.05 | 11000 | 4443 | 165 | 5.3 | 1.32 | 7323 | 2618 | 94.8 | 8.45 | 0.97 | 2311 | 1025 | 33.6 |
| 12.0 | *0.23* | *1700* | *242* | *6.4* | 9.0 | 1.12 | 4010 | 979 | 30.0 | 10.1 | 0.48 | 930 | 300 | 8.0 |
| 13.0 | *0.22* | *1396* | *198* | *5.2* | 9.8 | 0.71 | 2421 | 484 | 14.3 | 11.8 | 0.61 | 857 | 232 | 6.5 |
| 14.0 | *0.26* | *1389* | *209* | *5.5* | 10.2 | 0.35 | 1167 | 220 | 6.0 | 13.3 | 0.73 | 980 | 264 | 7.2 |
| 15.0 | *0.28* | *1425* | *201* | *4.9* | 11.2 | *0.28* | *1011* | *163* | *4.8* | 15.8 | *0.15* | *332* | *52* | *1.4* |
| 21.0 | *0.26* | *1330* | *187* | *4.1* | 13.5 | 0.72 | 1920 | 341 | 9.9 | 20.0 | *0.19* | *407* | *65* | *1.5* |
| 22.0 | *0.27* | *1310* | *181* | *4.0* | 14.7 | 0.37 | 921 | 165 | 4.4 | 20.5 | *0.16* | *329* | *48* | *1.2* |
| 23.0 | *0.23* | *1001* | *134* | *2.7* | 15.3 | *0.23* | *626* | *88* | *2.2* | 22.2 | *0.26* | *428* | *72* | *1.5* |
| 24.0 | 0.30 | 1081 | 125 | 2.8 | 15.8 | *0.22* | *587* | *77* | *2.1* |  |  |  |  |  |
| 25.0 | 0.37 | 1169 | 141 | 3.2 | 19.5 | *0.24* | *613* | *82* | *1.9* |  |  |  |  |  |
|  |  |  |  |  | 21.0 | 0.62 | 1541 | 231 | 4.1 |  |  |  |  |  |
|  |  |  |  |  | 23.2 | 0.58 | 1228 | 142 | 3.1 |  |  |  |  |  |
|  |  |  |  |  |  |  |  |  |  |  |  |  |  |  |
| 1,3-DCB (B) 3 h: 11.6 µg/L  1,3-DCB (B) 6 h: 10.3 µg/L  Total excreted volume: 4078 mL  Total excreted dose  3,5-DCK: 9186 µg  2,4-DCP: 2586 µg  3,5-DCP: 91 µg | | | | | 1,3-DCB (B) 3 h: 4.6 µg/L  1,3-DCB (B) 6 h: 4.9 µg/L  Total excreted volume: 3479 mL  Total excreted dose  3,5-DCK: 5594 µg  2,4-DCP: 1400 µg  3,5-DCP: 45 µg | | | | | 1,3-DCB (B) 3 h: 2.6 µg/L  1,3-DCB (B) 6 h: 3.6 µg/L  Total excreted volume: 2982 mL  Total excreted dose  3,5-DCK: 2226 µg  2,4-DCP: 788 µg  3,5-DCP: 22 µg | | | | |

| Volunteer 7 | | | | | | | | | | | | | | | |  |
| --- | --- | --- | --- | --- | --- | --- | --- | --- | --- | --- | --- | --- | --- | --- | --- | --- |
| 1.5 ppm 1,3-DCB | | | | | 0.7 ppm 1,3-DCB | | | | | 1.5 ppm 1,3-DCB + face mask | | | | | |  |
| time  [h] | crea.  [g/L] | 3,5-DCC  [µg/L] | 2,4-DCP  [µg/L] | 3,5-DCP  [µg/L] | time  [h] | crea.  [g/L] | 3,5-DCC  [µg/L] | 2,4-DCP  [µg/L] | 3,5-DCP  [µg/L | | time  [h] | crea.  [g/L] | 3,5-DCC  [µg/L] | 2,4-DCP  [µg/L] | 3,5-DCP  [µg/L | |
| - 1 | 1.78 | < 10 | < 10 | < 0.1 | - 1 | *5.59* | *891* | *112* | *5.5* | | - 0.8 | *3.36* | *526* | *70* | *2.4* | |
| 3.75 | 1.63 | 11324 | 4173 | 169 | 6.8 | *4.96* | *22270* | *5616* | *324* | | 0.7 | *3.05* | *672* | *117* | *4.3* | |
| 7.0 | 1.91 | 18026 | 6100 | 253 | 10.0 | 2.44 | 8725 | 1615 | 66.8 | | 6.5 | 2.77 | 1824 | 523 | 20.6 | |
| 8.7 | 0.80 | 6829 | 1342 | 50.4 | 12.2 | 1.07 | 2876 | 297 | 15.3 | | 9.0 | 0.85 | 774 | 168 | 5.2 | |
| 13.5 | 1.52 | 8022 | 1290 | 48.6 | 15.6 | 1.54 | 3724 | 563 | 16.7 | | 10.1 | 0.60 | 497 | 83 | 2.4 | |
| 18.3 | 1.74 | 5904 | 802 | 25.5 | 24.3 | 1.30 | 1990 | 240 | 6.8 | | 14.0 | 1.74 | 971 | 192 | 4.8 | |
| 24.3 | 1.01 | 2910 | 368 | 9.4 |  |  |  |  |  | | 23.8 | 2.19 | 853 | 141 | 3.5 | |
|  |  |  |  |  |  |  |  |  |  | |  |  |  |  |  | |
| 1,3-DCB (B) 3 h: 9.6 µg/L  1,3-DCB (B) 6 h: 9.2 µg/L  Total excreted volume: 1594 mL  Total excreted dose  3,5-DCK: 12406 µg  2,4-DCP: 2886 µg  3,5-DCP: 112 µg | | | | | 1,3-DCB (B) 3 h: 7.0 µg/L  1,3-DCB (B) 6 h: 6.7 µg/L  Total excreted volume: 1293 mL  Total excreted dose  3,5-DCK: 8105 µg  2,4-DCP: 1642 µg  3,5-DCP: 81 µg | | | | | 1,3-DCB (B) 3 h: 0.9 µg/L  1,3-DCB (B) 6 h: 0.8 µg/L  Total excreted volume: 1366 mL  Total excreted dose  3,5-DCK: 1280 µg  2,4-DCP: 276 µg  3,5-DCP: 9 µg | | | | | |  |

| Volunteer 8 | | | | | | | | | | | | | | |  |
| --- | --- | --- | --- | --- | --- | --- | --- | --- | --- | --- | --- | --- | --- | --- | --- |
| 1.5 ppm 1,3-DCB | | | | | 0.7 ppm 1,3-DCB | | | | | 1.5 ppm 1,3-DCB + face mask | | | | |  |
| time  [h] | crea.  [g/L] | 3,5-DCC  [µg/L] | 2,4-DCP  [µg/L] | 3,5-DCP  [µg/L] | time  [h] | crea.  [g/L] | 3,5-DCC  [µg/L] | 2,4-DCP  [µg/L] | 3,5-DCP  [µg/L | time  [h] | crea.  [g/L] | 3,5-DCC  [µg/L] | 2,4-DCP  [µg/L] | 3,5-DCP  [µg/L | |
| - 0.5 | 2.00 | < 10 | < 10 | < 0.1 | - 1 | 1.89 | 368 | 57 | 1.8 | - 0.8 | 2.31 | 517 | 97 | 2.4 | |
| 2.8 | 0.53 | 3579 | 1532 | 64.3 | 2.2 | 0.88 | 2511 | 962 | 38.1 | 2.4 | 2.05 | 743 | 202 | 7.5 | |
| 7.5 | 1.59 | 21374 | 7013 | 312 | 7.2 | 1.23 | 6588 | 2056 | 80.9 | 8.8 | *3.20* | *2614* | *793* | *29.0* | |
| 10.75 | 1.04 | 10902 | 2408 | 86.9 | 9.3 | 0.32 | 1553 | 329 | 10.0 | 11.75 | 2.80 | 2179 | 430 | 14.7 | |
| 12.5 | 0.44 | 3429 | 583 | 18.4 | 11.3 | 0.61 | 2181 | 393 | 10.8 | 14.25 | 2.33 | 1553 | 242 | 7.6 | |
| 21.5 | 1.54 | 8441 | 1231 | 36.1 | 14.7 | 2.20 | 5383 | 1010 | 25.6 | 23.0 | 2.07 | 1141 | 186 | 4.3 | |
|  |  |  |  |  | 23.3 | 2.01 | 3513 | 549 | 12.0 |  |  |  |  |  | |
|  |  |  |  |  |  |  |  |  |  |  |  |  |  |  | |
| 1,3-DCB (B) 3 h: 10.1 µg/L  1,3-DCB (B) 6 h: 6.0 µg/L  Total excreted volume: 1702 mL  Total excreted dose  3,5-DCK: 15244 µg  2,4-DCP: 3979 µg  3,5-DCP: 159 µg | | | | | 1,3-DCB (B) 3 h: 4.4 µg/L  1,3-DCB (B) 6 h: 4.8 µg/L  Total excreted volume: 1804 mL  Total excreted dose  3,5-DCK: 6056 µg  2,4-DCP: 1584 µg  3,5-DCP: 56 µg | | | | | 1,3-DCB (B) 3 h: 1.3 µg/L  1,3-DCB (B) 6 h: 1.4 µg/L  Total excreted volume: 1316 mL  Total excreted dose  3,5-DCK: 2245 µg  2,4-DCP: 513 µg  3,5-DCP: 17 µg | | | | |  |

| Volunteer 9 | | | | | | | | | | | | | | |
| --- | --- | --- | --- | --- | --- | --- | --- | --- | --- | --- | --- | --- | --- | --- |
| 1.5 ppm 1,3-DCB | | | | | 0.7 ppm 1,3-DCB | | | | | 1.5 ppm 1,3-DCB + face mask | | | | |
| time  [h] | crea.  [g/L] | 3,5-DCC  [µg/L] | 2,4-DCP  [µg/L] | 3,5-DCP  [µg/L] | time  [h] | crea.  [g/L] | 3,5-DCC  [µg/L] | 2,4-DCP  [µg/L] | 3,5-DCP  [µg/L | time  [h] | crea.  [g/L] | 3,5-DCC  [µg/L] | 2,4-DCP  [µg/L] | 3,5-DCP  [µg/L |
| - 0.25 | 2.07 | < 10 | < 10 | < 0.1 | - 0.3 | 2.51 | 213 | 28 | 0.9 | - 1 | 2.36 | 153 | 19 | 0.7 |
| 4.25 | 0.95 | 8017 | 2298 | 125 | 3.3 | 1.04 | 2481 | 983 | 41.7 | 5.0 | 1.75 | 884 | 214 | 10.1 |
| 7.25 | 2.55 | 38083 | 12583 | 644 | 6.8 | 2.75 | 12385 | 4887 | 194 | 6.25 | 2.49 | 1776 | 499 | 22.6 |
| 9.25 | 0.62 | 8131 | 2109 | 78.6 | 10.3 | 1.11 | 4224 | 1285 | 39.0 | 10.25 | 1.00 | 759 | 153 | 5.9 |
| 10.25 | 0.19 | 2242 | 355 | 15.0 | 13.0 | 0.84 | 2195 | 483 | 13.9 | 14.8 | 1.36 | 763 | 143 | 3.8 |
| 12.3 | 1.20 | 9430 | 1617 | 67.7 | 14.6 | 0.31 | 807 | 149 | 3.5 | 16.25 | 0.49 | 283 | 71 | 1.1 |
| 14.1 | 0.30 | 2355 | 307 | 11.9 | 23.1 | 1.75 | 2862 | 565 | 13.9 | 22.0 | 0.69 | 332 | 13 | 1.3 |
| 15.3 | 0.49 | 2976 | 389 | 15.5 |  |  |  |  |  | 23.25 | 0.82 | 318 | 19 | 1.3 |
| 22.25 | 1.65 | 7901 | 1103 | 35.6 |  |  |  |  |  |  |  |  |  |  |
|  |  |  |  |  |  |  |  |  |  |  |  |  |  |  |
| 1,3-DCB (B) 3 h: 8.5 µg/L  1,3-DCB (B) 6 h: 8.5 µg/L  Total excreted volume: 2036 mL  Total excreted dose  3,5-DCK: 13960 µg  2,4-DCP: 3360 µg  3,5-DCP: 156 µg | | | | | 1,3-DCB (B) 3 h: 6.5 µg/L  1,3-DCB (B) 6 h: 7.3 µg/L  Total excreted volume: 1640 mL  Total excreted dose  3,5-DCK: 4808 µg  2,4-DCP: 1479 µg  3,5-DCP: 51 µg | | | | | 1,3-DCB (B) 3 h: 1.2 µg/L  1,3-DCB (B) 6 h: 0.8 µg/L  Total excreted volume: 1760 mL  Total excreted dose  3,5-DCK: 1011 µg  2,4-DCP: 184 µg  3,5-DCP: 7 µg | | | | |

| Volunteer 10 | | | | | | | | | | | | | | |  |
| --- | --- | --- | --- | --- | --- | --- | --- | --- | --- | --- | --- | --- | --- | --- | --- |
| 1.5 ppm 1,3-DCB | | | | | 0.7 ppm 1,3-DCB | | | | | 1.5 ppm 1,3-DCB + face mask | | | | |  |
| time  [h] | crea.  [g/L] | 3,5-DCC  [µg/L] | 2,4-DCP  [µg/L] | 3,5-DCP  [µg/L] | time  [h] | crea.  [g/L] | 3,5-DCC  [µg/L] | 2,4-DCP  [µg/L] | 3,5-DCP  [µg/L | time  [h] | crea.  [g/L] | 3,5-DCC  [µg/L] | 2,4-DCP  [µg/L] | 3,5-DCP  [µg/L | |
| - 0.25 | 1.00 | < 10 | < 10 | < 0.1 | - 0.5 | 1.49 | 115 | 22 | 0.5 | - 0.8 | 1.69 | 118 | 11 | 0.4 | |
| 1.75 | 0.56 | 3577 | 1012 | 71.3 | 0.3 | 0.59 | 1276 | 359 | 20.7 | 2.25 | 0.84 | 633 | 144 | 7.2 | |
| 6.5 | 1.17 | 19636 | 6383 | 308 | 2.2 | 0.32 | 1529 | 383 | 20.9 | 6.75 | 1.83 | 2926 | 732 | 35.5 | |
| 9.0 | 1.13 | 18157 | 5067 | 267 | 6.0 | 0.72 | 5017 | 1418 | 62.2 | 8.66 | 0.83 | 1359 | 373 | 13.0 | |
| 10.5 | 0.89 | 11164 | 2676 | 117 | 10.5 | 1.44 | 5746 | 1426 | 47.1 | 9.33 | *0.19* | *419* | *85* | *2.6* | |
| 15.1 | 1.67 | 14764 | 2612 | 111 | 11.5 | 1.48 | 4281 | 858 | 29.0 | 13.5 | 1.31 | 48 | 232 | 6.1 | |
| 18.0 | 1.99 | 13614 | 2532 | 88.4 | 22.2 | 2.41 | 4998 | 844 | 25.6 | 20.75 | 1.65 | 993 | 191 | 4.7 | |
| 22.3 | 2.10 | 11231 | 1548 | 55.9 |  |  |  |  |  | 22.66 | 1.71 | 853 | 183 | 4.6 | |
|  |  |  |  |  |  |  |  |  |  |  |  |  |  |  | |
| 1,3-DCB (B) 3 h: 9.9 µg/L  1,3-DCB (B) 6 h: 11.4 µg/L  Total excreted volume: 1382 mL  Total excreted dose  3,5-DCK: 16835 µg  2,4-DCP: 4132 µg  3,5-DCP: 198 µg | | | | | 1,3-DCB (B) 3 h: 2.5 µg/L  1,3-DCB (B) 6 h: 5.9 µg/L  Total excreted volume: 1482 mL  Total excreted dose  3,5-DCK: 5400 µg  2,4-DCP: 1318 µg  3,5-DCP: 54 µg | | | | | 1,3-DCB (B) 3 h: 2.6 µg/L  1,3-DCB (B) 6 h: 1.6 µg/L  Total excreted volume: 1766 mL  Total excreted dose  3,5-DCK: 1646 µg  2,4-DCP: 440 µg  3,5-DCP: 16 µg | | | | |  |
